# Establishment of persistent enteric mycobacterial infection following streptomycin pre-treatment

**DOI:** 10.1186/s13099-023-00573-w

**Published:** 2023-10-03

**Authors:** Shannon C. Duffy, Andréanne Lupien, Youssef Elhaji, Mina Farag, Victoria Marcus, Marcel A. Behr

**Affiliations:** 1 Department of Microbiology and Immunology, McGill University, Montreal, QC Canada; 2 McGill International TB Centre, Montreal, QC Canada; 3 The Infectious Diseases and Immunity in Global Health Program, The Research Institute of the McGill University Health Centre, Montreal, QC Canada; 4 Department of Medicine, McGill University, Montreal, QC Canada; 5 Diagnostic Genomic Division, Department of Laboratory Medicine and Pathology, Hamad Medical Corporation, Doha, Qatar; 6 Department of Pathology, McGill University, Montreal, QC Canada; 7 Department of Laboratory Medicine, Division of Pathology, McGill University Health Center, Montreal, QC Canada

**Keywords:** *Mycobacterium avium* subsp. *paratuberculosis*, Paratuberculosis, Crohn’s disease, Mouse models, *Mycobacterium avium* subsp. *hominissuis*, *Mycobacterium bovis*, *Mycobacterium orygis*

## Abstract

*Mycobacterium avium* subsp. *paratuberculosis* (*MAP*) is the causative agent of paratuberculosis, a chronic gastrointestinal disease affecting ruminants. This disease remains widespread in part due to the limitations of available diagnostics and vaccines. A representative small animal model of disease could act as a valuable tool for studying its pathogenesis and to develop new methods for paratuberculosis control, but current models are lacking. Streptomycin pre-treatment can reduce colonization resistance and has previously been shown to improve enteric infection in a *Salmonella* model. Here, we investigated whether streptomycin pre-treatment of mice followed by *MAP* gavage could act as a model of paratuberculosis which mimics the natural route of infection and disease development in ruminants. The infection outcomes of *MAP* were compared to *M. avium* subsp. *hominissuis* (*MAH*), an environmental mycobacterium, and *M. bovis* and *M. orygis*, two tuberculous mycobacteria. Streptomycin pre-treatment was shown to consistently improve bacterial infection post-oral inoculation. This model led to chronic *MAP* infection of the intestines and mesenteric lymph nodes (MLNs) up to 24-weeks post-gavage, however there was no evidence of inflammation or disease. These infection outcomes were found to be specific to *MAP.* When the model was applied to a bacterium of lesser virulence *MAH*, the infection was comparatively transient. Mice infected with bacteria of greater virulence, *M. bovis* or *M. orygis*, developed chronic intestinal and MLN infection with pulmonary disease similar to zoonotic TB. Our findings suggest that a streptomycin pre-treatment mouse model could be applied to future studies to improve enteric infection with *MAP* and to investigate other modifications underlying *MAP* enteritis.

**Supplementary Information:**

The online version contains supplementary material available at 10.1186/s13099-023-00573-w.

## Introduction

*Mycobacterium avium* subsp. *paratuberculosis* (*MAP*) is the cause of a chronic gastrointestinal disease called paratuberculosis which affects ruminants such as cattle, sheep, goats, and deer [1]. Post- *MAP* infection, animals enter an initial subclinical period lasting 2–5 years which is followed by clinical disease characterized by diarrhea, wasting, and eventual death [2]. Control of paratuberculosis relies on a test and cull strategy to remove infected animals from the herd, however its efficacy is constrained by several factors such as the limitations of *MAP* diagnostics [3, 4]. Paratuberculosis therefore remains widespread with global prevalence estimates ranging from 10–70% [3]. This disease poses a major economic burden in many countries, such as in the United States, where it is associated with a cost of $198 million per year [5]. In addition, *MAP* may also pose a threat to public health if, as hypothesized, it is etiologically linked to Crohn’s disease [6].

*MAP* is a nontuberculous mycobacterium which emerged from *M. avium* subsp. *hominissuis* (*MAH*), an environmental species which can cause opportunistic disease in humans and pigs [7, 8]. *MAP* is an obligate intracellular pathogen of intestinal macrophages [9]. It will invade the intestines through M cells in Peyer’s patches and infect macrophages found within the lamina propria [9]. The infected macrophages may remain local or travel to the mesenteric lymph nodes (MLNs) where *MAP* can also persist [10]. Diseased animals will develop granulomatous enteritis with a thickening of the intestinal wall, primarily in the terminal ileum, accompanied by inflamed MLNs [11]. *MAP* is shed into the feces allowing other animals to be infected via the fecal to oral route [3]. However, *MAP* may also be acquired from the environment due to the ability of the bacteria to persist for long periods of time in water and soil [12, 13].

To study *MAP* pathogenesis and develop new methods for paratuberculosis control, a small animal model could be a valuable tool. Bovine models, while offering the obvious benefit of being a natural *MAP* host, are often an impractical choice due to their cost, space, and personnel training requirements. Mouse models alternatively offer several benefits including reduced cost, increased accessibility, and availability of a variety of defined and well-characterized mouse strains. Although intraperitoneal injection of mice with *MAP* leads to reproducible infection of the spleen and liver, it does not mimic the natural route of infection [14]. An oral infection model would match the natural route; however, gavage of *MAP* can lead to inconsistent infection and does not result in intestinal disease [15, 16]. This phenomenon has also been observed with other known enteropathogens such as enteropathogenic and enterohemorrhagic *Escherichia coli* [17, 18]. In the case of *Salmonella enteria* serovar Typhimurium, gavage infections in mice lead to systemic dissemination rather than local disease in the intestine. However, Barthel *et al.* found that if the mice were pre-treated with streptomycin to reduce colonization resistance posed by gut microbes prior to gavage with a streptomycin-resistant strain of *S. enterica* serovar Typhimurium, intestinal infection does occur with disease which more closely mimics the enterocolitis observed in humans [19].

Here, we investigated whether an oral streptomycin pre-treatment model of *MAP* infection could act as a representative mouse model of *MAP*-induced intestinal infection and disease. To compare how our *MAP* results compared with other mycobacteria, we applied the same infection strategy to *MAH,* a mycobacterium of lesser virulence*,* which is primarily environmental but has been shown to cause opportunistic infections in immunocompromised humans and pigs [20]. The model was also applied to 2 mycobacteria of greater virulence (*M. bovis*, *M. orygis*) which are causes of bovine and zoonotic TB and are suggested to infect humans through drinking unpasteurized milk [21, 22]. Overall, we determined that following streptomycin pre-treatment, *MAP* resulted in chronic intestinal infection with no evidence of intestinal disease, unlike *MAH* which infected only transiently and *M. bovis/M. orygis* which disseminated to the lungs and caused pulmonary disease.

## Materials and methods

### Media

All mycobacterial strains were grown in Middlebrook 7H9 broth (Difco Laboratories, Detroit, MI) with 0.2% glycerol, 0.1% Tween 80, and 10% albumin-dextrose-catalase (Becton, Dickinson and Co., Sparks, MD) with rotation at 37 °C. For colony isolation, the bacteria were plated on Middlebrook 7H10 medium supplemented with 10% oleic acid-albumin-dextrose-catalase (Becton, Dickinson and Co.). To grow *MAP*, 0.1% mycobactin J (Allied Monitor, Fayette MO) was added to liquid and solid media. The antibiotics kanamycin (50 µg/mL), streptomycin (50 µg/mL), or PANTA antibiotic mixture (Becton, Dickinson and Co.) were included in the media when required.

### Generation of streptomycin-resistant strains

To generate streptomycin-resistant (strep-R) strains of *MAP* K10, *MAH* 104, *M. bovis* Ravanel and *M. orygis* 51145, a K43R mutation was introduced into the *rpsL* gene of each species using oligo-mediated recombineering as previously described with minor modifications [23]. This mutation is known to confer resistance to streptomycin [24]. In brief, the pNit::ET plasmid (from Kenan Murphy – Addgene plasmid #107692) was first introduced into each strain and selected on kanamycin-containing media. The plasmid was confirmed by PCR of the kanamycin cassette (Additional file 1: Table S1). To introduce the K43R mutation in *MAP, M. bovis* and *M. orygis*, 30 mL cultures containing pNit::ET were grown to log phase in 7H9 media with kanamycin and then diluted to an OD_600_ of 0.1. When cultures reached an OD_600_ of 0.8, 30 µL of a 1000X stock of isovaleronitrile was added to stimulate the pNit::ET plasmid. When cultures reached an OD_600_ of 1.0, 3 mL of 2 M glycine was added to the culture. The next day, the cultures were washed 3 times in 10% glycerol. The cells were resuspended in 1 mL 10% glycerol and 200 µL of cells were transferred to a 2 mm gap electroporation cuvette (Fisherbrand, Waltham, MA) containing 1 µg of a 70-mer oligo designed to introduce the K43R mutation (Additional file 1: Table S1). The cells were electroporated using the following settings: 2.5 kV, 1,000 Ω, and 25 µF. The cells were then transferred to 3 mL of 7H9 and incubated at 37 °C. After 5 days (*MAP*) or 2 days (*M. bovis, M. orygis*), the cultures were recovered on 7H10 agar with streptomycin.

To generate the *rpsL* mutation in *MAH*, the same procedure was followed except for the following modifications: the starting culture was 50 mL, 50 µL of isovaleronitrile was used in stimulation, 7.5 mL of 2 M glycine was added prior to electroporation, washes were performed with pre-warmed 10% glycerol with 2 M sucrose, cells were resuspended in 800 µL 7H9 with 2 M sucrose prior to recovery, and cells were recovered after 1 day.

Colonies which grew on streptomycin were grown up and screened for the K43R mutation by PCR of the *rpsL* gene followed by Sanger sequencing (Additional file 1: Table S1). Sequences were visualized using Geneious Prime (version 2022.1.1).

### Whole genome sequencing and analysis

Genomic DNA was extracted from strep-R *MAP* and the parental strain using the QIAamp UCP pathogen mini kit (Qiagen, Hilden, Germany) according to manufacturer’s instructions. Paired end sequencing libraries were prepared using the S4 reagent kit (Illumina, San Diego, CA) and shotgun sequencing was performed using the NovaSeq 6000 S4 PE150–35 M reads (Illumina). The sequence was aligned to the *MAP* K10 reference genome (NC_002944.2) using BWA-MEM [25]. The reads were sorted using SAMtools and visualized using Integrative Genomics Viewer (IGV) [26, 27].

The strep-R *MAP* sequence was analyzed for deletions and for SNPs outside of the engineered K43R mutation in *rpsL.* Duplicate reads in sorted BAM files were removed using Picard (http://broadinstitute.github.io/picard). Variant calling was done using Freebayes v1.3.6 with mapping quality 60, minimum read coverage 10, and minimum allele frequency of 0.5 [28]. Variant calls were annotated using SNPEff v.4.3 [29]. Variants identified in strep-R *MAP* were compared with the parental strain to identify unique variants.

### pH experiment

The gastric pH of a mouse is between 3.0 (fed) and 4.0 (after fasting) [30]. To quantify the amount of *MAP* potentially lost due to the acidic environment of the stomach, strep-R *MAP* was grown to an OD_600_ of 1.0 (~ 2 × 10^8^ colony-forming units (CFU)/mL) in 7H9 media. The culture was then split and resuspended in PBS with 0.1% Tween-80 at either a pH of 7.0 or 3.0 and incubated at 37 °C for 1, 2, or 4 h. At the indicated timepoint, the cultures were spun down and resuspended in 7H9 then serially diluted and plated on 7H10 agar.

### Animals

Mice were housed in a pathogen-free environment at the Research Institute of the McGill University Health Centre (RI-MUHC). All animal experiments were in accordance with the guidelines of the Canadian Council on Animal Care (CCAC) and all protocols were approved by the animal resource division of the RI-MUHC. C57BL/6 and BALB/c mice were purchased from The Jackson Laboratory (Bar Harbor, ME) at 7-weeks of age and quarantined in-house for 1 week prior to infections. All mouse infections were sex and age matched.

### *MAP* mouse infection model

Infection outcomes were compared using a strep-R *MAP* inoculum of 10^8^ or 10^9^ CFU. Bacterial inoculum was prepared by growing strep-R *MAP* to an OD_600_ of 0.5 (~ 1 × 10^8^ CFU/mL) in 7H9 with streptomycin, washing the cells once in PBS with 0.01% Tween-80, and then resuspending the bacteria in either 1/5 or 1/50 the original volume in PBS with 0.01% Tween-80 to generate a stock of 5 × 10^8^ CFU/mL or 5 × 10^9^ CFU/mL. Prior to all gavage steps, mice were fasted of food and water for 3 h. The first day, mice were given 200 µL of streptomycin (100 mg/mL) by oral gavage. Twenty-four hours later mice were orally inoculated with 200 µL of the prepared strep-R *MAP* stock (equivalent to a dose of 10^8^ or 10^9^ CFUs). Mice given the higher dose were given a second 10^9^ CFU strep-R *MAP* infection by oral gavage the following day. When indicated, mice were given 100 µL of 3% sodium bicarbonate 30 minutes [31] prior to oral gavage of strep-R *MAP.* At the indicated timepoints, mice were sacrificed, and the small intestine, large intestine, MLNs, spleen, liver, and lungs were taken for quantification of organ CFUs. The small intestine, large intestine, and MLNs were also sent for histopathology assessment.

### *MAH* murine infection model

A 5 × 10^9^ CFU/mL bacterial stock of strep-R *MAH* was prepared by growing the bacteria to an OD_600_ of 0.5 (~ 1 × 10^8^ CFU/mL). The culture was washed once in PBS with 0.01% Tween-80 and then resuspended in 1/50 of the original volume in PBS with 0.01% Tween-80. Mice were fasted of food and water for 3 h prior to all oral gavage steps. Mice were given 200 µL of streptomycin (100 mg/mL) followed by two consecutive doses of 200 µL of strep-R *MAH* (5 × 10^9^ CFU/mL) at 24-hour intervals. Mice infected with strep-R *MAH* were euthanized 48-hours, 4-, 8-, 12-, and 24-weeks post-infection and the small intestine, large intestine, and MLNs were assessed for CFUs.

### *M. bovis and M. orygis *mouse infection model

All *M. bovis* and *M. orygis* experiments took place in the containment level 3 facilities at the RI-MUHC as a fully virulent *M. bovis* strain (*M. bovis* Ravanel) was used rather than an *M. bovis* BCG vaccine strain. *M. orygis* is a cause of zoonotic TB in people in or migrating from South Asia [21], and a clinical isolate collected in Canada (*M. orygis* 51145) was used [32]. A 5 × 10^9^ CFU/mL bacterial stock of strep-R *M. bovis*/*M. orygis* was prepared by first growing strep-R *M. bovis*/*M. orygis* in 7H9 to an OD_600_ of 0.5 (~ 5 × 10^7^ CFU/mL). The culture was washed once in PBS with 0.01% Tween-80 and then resuspended in 1/100 of the original volume in PBS with 0.01% Tween-80. Mice were fasted of food and water for 3 h prior to all oral gavage steps. Mice were given either 200 µL of streptomycin (100 mg/mL) or no pre-treatment followed by two consecutive doses of strep-R *M. bovis*/*M. orygis* spaced 24-hours apart. Due to the recognized virulence of *M. bovis*/*M. orygis* in mice [33, 34], infected mice were weighed and monitored for survival throughout the 24-week experiment. Mice were euthanized at 4- and 24- weeks post-infection and the small intestine, large intestine, MLNs, and lungs were assessed for CFUs and histopathology.

### Organ CFU quantification

At each timepoint, the small and large intestines were excised, separated, and cut open longitudinally. The fecal matter and mucus were removed mechanically using the flat end of curved tweezers, and the organs were washed 3 times in PBS with 0.01% Tween-80 before being placed in 1 mL 7H9. The intestines were processed in this way to identify *MAP* CFUs that had invaded into the organ tissue and to avoid CFUs passing through the intestines as a result of the inoculation at the earlier timepoints. The spleens, livers, and lungs were placed directly in 1 mL 7H9. The organs were then homogenized with an Omni Tissue Homogenizer TH (Omni International, Kennesaw, GA) for 45 s. Finally, the MLN chain was directly pushed through a 70 μm sterile cell strainer (Fisher Scientific, Waltham, MA) into 1 mL 7H9. The resulting homogenates for all collected organs were serially diluted in 7H9 and plated on 7H10 containing PANTA to quantify CFUs. If no colonies were counted on the lowest dilutions plated, the organ was assigned the limit of detection (LOD).

### F57 real-time PCR of fecal pellets

DNA was extracted from fecal pellets of C57BL/6 and BALB/c mice that were uninfected or 12-weeks post-*MAP* infection using the QIAmp PowerFecal Pro DNA kit (Qiagen, Hilden, Germany) according to the manufacturer’s instructions. The *MAP* K10 genome is 4,829,781 base pairs which corresponds to ~ 5.3 fg per copy [35]. A standard curve for qPCR was prepared from 1 × 10^7^ genome equivalents (ge) (53 ng) to 1 ge (5.3 fg). A Maxima SYBR Green/ROX real-time PCR assay (Thermo Fisher Scientific, Waltham, MA) was performed using primers for the single copy gene F57 (Additional file 1: Table S1) following the manufacturer’s instructions.

### Histopathology

Small intestines and large intestines from all infected and uninfected control mice, mesentery from *MAP-*infected and uninfected mice, and lungs from *M. bovis-*infected, *M. orygis-*infected, and uninfected mice were sent to the histology core at McGill University. The organs were paraffin-embedded and 4 μm sections were cut and stained by hematoxylin and eosin (H&E) staining. Positive control slides of enteritis were generously provided from Dr. Laura Sly’s lab who has a model of spontaneous enteritis in SHIP^−/−^ mice [36]. All slides were reviewed by a pathologist at the MUHC. All slides were photographed with a Nikon Eclipse NI microscope.

### Lipocalin-2 ELISA

Fecal pellets were collected from infected and uninfected control mice and stored at −80 °C until processed. Fecal pellets were weighed and placed in a 1.5 mL screwcap tube. PBS with 0.1% Tween-20 was added at a volume of 10 μl per 1 mg of feces. The tubes were vortexed at maximum speed for 20 min and then spun down in a microcentrifuge at 12,000 x g for 10 min at 4 °C. The supernatant was transferred into a new screwcap tube and stored at −20 °C until ready for ELISA of lipocalin-2, a broad marker of intestinal inflammation in mice [37]. The assay was performed using the mouse lipocalin-2/NGAL DuoSet ELISA (Bio-techne, Minneapolis, MO) and DuoSet ELISA ancillary reagent kit (Bio-techne) according to the manufacturer’s instructions.

### Statistics

Statistical analyses were conducted with GraphPad Prism (version 9.3.1). Grouped data are graphed as individual datapoints with the sample median. Multiple groups comparisons were performed using a two-way ANOVA with Sidak’s multiple comparisons test. To compare 2 groups, an unpaired t-test was used. Analysis of pooled organ data was performed using a Mann-Whitney test.

## Results

### Streptomycin pre-treatment increases *MAP* bacterial burden following oral infection

**Fig. 1:**
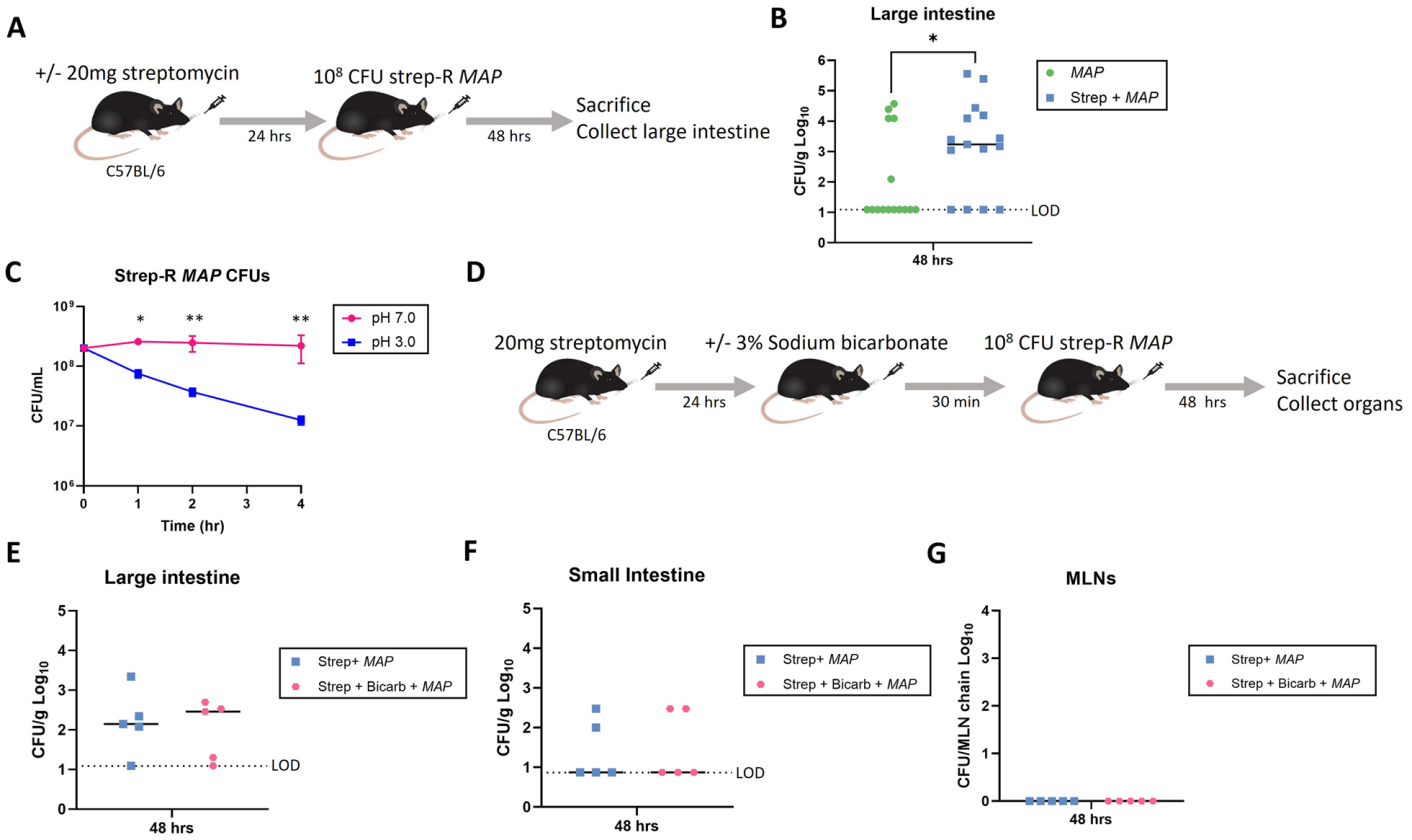
The effect of pre-treatment with streptomycin and sodium bicarbonate on *MAP* infection. **A** C57BL/6 mice were given 20 mg of streptomycin or no pre-treatment followed by an oral gavage of 10^8^ CFU strep-R *MAP* 24-hours later. Mice were sacrificed and the large intestines were assessed for CFUs 48-hours post-infection. **B** Large intestine CFUs were compared between mice given streptomycin pre-treatment or no pre-treatment (*p < 0.05). **C** Strep-R *MAP* was subject to a pH of 7.0 or 3.0 for 1, 2, or 4 hours. The CFUs following incubation at either pH was plotted over time (*p < 0.05, **p < 0.01). **D** Mice were orally given 20 mg of streptomycin. The next day half of the mice were given sodium bicarbonate followed by a gavage of 10^8^ CFU strep-R *MAP* in all mice 30 minutes later. Mice were sacrificed and their organs were assessed for CFUs 48-hours post-infection. **E**–**G** The* MAP *CFUs of the large intestine (**E**), small intestine (**F**), and MLNs (**G**) were compared between mice given sodium bicarbonate and streptomycin pre-treatment or only streptomycin pre-treatment

A strep-R *MAP* strain was generated by introducing a K43R mutation into the *rpsL* gene via oligo-mediated recombineering. The SNP introduction was confirmed via Sanger sequencing (Additional file 1: Fig. S1A) and the strain was shown to grow on 7H10 agar with 50 µg/mL streptomycin (Additional file 1: Fig. S1B). Whole-genome sequencing was performed on the strep-R *MAP* and parent strain to determine whether any off-target mutations occurred in the generation of strep-R *MAP*. No deletions were detected and only 1 additional point mutation was detected in *mtrB*, a gene which makes up a two-component regulatory system. The *mtrAB* system has previously been associated with multidrug resistance in *M. avium*, therefore it is possible that this is a compensatory mutation [38]. To test whether streptomycin pre-treatment would improve infection of *MAP* following oral gavage, C57BL/6 mice were given 20 mg of streptomycin or no pre-treatment followed by a dose of 10^8^ CFU strep-R *MAP* 24-hours later. After 48 h, mice were euthanized and the organ CFUs were compared between the 2 groups (Fig. 1A). The mean strep-R *MAP* CFUs were ~ 14X greater (*p = 0.04) in the large intestine in mice that received streptomycin pre-treatment (Fig. 1B).

### Increasing gastric pH does not improve *MAP* infection following oral inoculation

In order to determine whether *MAP* intestinal load could be further increased following streptomycin pre-treatment, the effect of the acidic gastric pH of the mouse stomach on *MAP* death following gavage was tested. The effect of pH on *MAP* survival was tested both in vitro and *in vivo.* Strep-R *MAP* was grown to an OD_600_ of 1.0 (~ 2 × 10^8^ CFU) and then transferred to PBS at a pH of either 7.0 or 3.0. There were significantly fewer CFUs in cultures exposed to an acidic pH after 1- (*p = 0.019), 2- (**p = 0.009) and 4-hours (**p = 0.009). One hour in PBS at a pH of 3.0 decreased the number of viable strep-R *MAP* by ~ 0.5-log and 4 h decreased the number of viable strep-R *MAP* by > 1-log (Fig. 1C).

To investigate whether increasing the mouse gastric pH would therefore improve *MAP* infection post-gavage, mice were administered sodium bicarbonate prior to gavage of strep-R *MAP*. Mice were pre-treated with 20 mg of streptomycin and then 24-hours later half of the mice were given 3% sodium bicarbonate 30 minutes [31] before all mice were given 1 × 10^8^ CFU strep-R *MAP* (Fig. 1D). After 48 h, there were no differences in bacterial burden found between mice that received sodium bicarbonate and those that did not in the large intestine (Fig. 1E), small intestine (Fig. 1F) or MLNs (Fig. 1G). This indicated that although a low pH reduced strep-R *MAP* CFUs in vitro, increasing gastric pH in vivo did not improve strep-R *MAP* organ infection post-gavage and therefore was not performed in later experiments.

### Two consecutive oral doses of strep-R *MAP* led to infection of the mesenteric lymph nodes

**Fig. 2:**
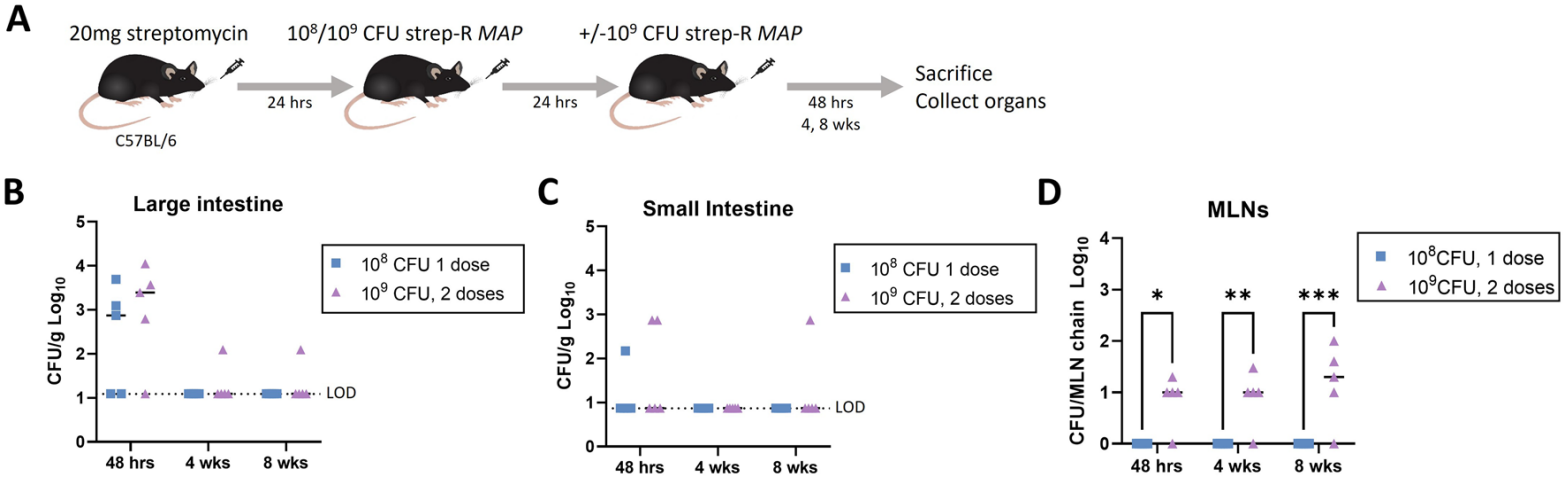
Comparison of a single dose of 10^8^ CFU strep-R *MAP* and 2 consecutive doses of 10^9^ CFU strep-R MAP. **A** Mice were pre-treated with 20 mg of streptomycin. The following day, mice were given either 10^8^ or 10^9^ CFU strep-R *MAP*. Mice that were inoculated with the higher dose were given a second dose of 10^9^ CFU strep-R *MAP* 24-hours later. Mice were euthanized 48-hours, 4-weeks, or 8-weeks post-infection and the organs were assessed for CFUs. **B**–**D** Strep-R *MAP* CFUs were compared between the lower and higher dose infection strategies in the large intestine (**B**), small intestine (**C**), and MLNs (**D**) (*p < 0.05, **p < 0.01, ***p < 0.001)

The effect of increasing the dosage of strep-R *MAP* organ infection was next evaluated. C57BL/6 mice were given streptomycin pre-treatment followed either by a single dose of 1 × 10^8^ CFU strep-R *MAP* or 2 doses of 1 × 10^9^ CFU given 24-hours apart (Fig. 2A). Organ CFUs were assessed at 48-hours, 4-, and 8-weeks post-infection. No differences were observed between the doses in infection of the large intestine (Fig. 2B) or small intestine (Fig. 2C). However, only mice that received the 2 high doses of strep-R *MAP* had CFUs in the MLNs (Fig. 2D), suggesting that a greater dose is required for *MAP* migration to the lymph nodes. This was significant at 48-hours (*p = 0.013) 4- (**p = 0.009) and 8-weeks (***p = 0.001) post-infection. At 48-hours post-infection, strep-R *MAP* CFUs in the higher dose group were greatest in the large intestine (~ 10^3^-10^4^ CFU/g) and MLNs (~ 10^1^ CFU/MLN chain). Later at 4- and 8-weeks post-infection, strep-R *MAP* CFUs were primarily restricted to the MLNs (~ 10^1^-10^2^ CFU/MLN chain). This persistence of *MAP* within the MLNs is consistent with the known course following natural infection [10]. The higher dose infection model was therefore chosen for the remaining mouse experiments.

### The streptomycin pre-treatment *MAP* model leads to chronic infection without disease

**Fig. 3:**
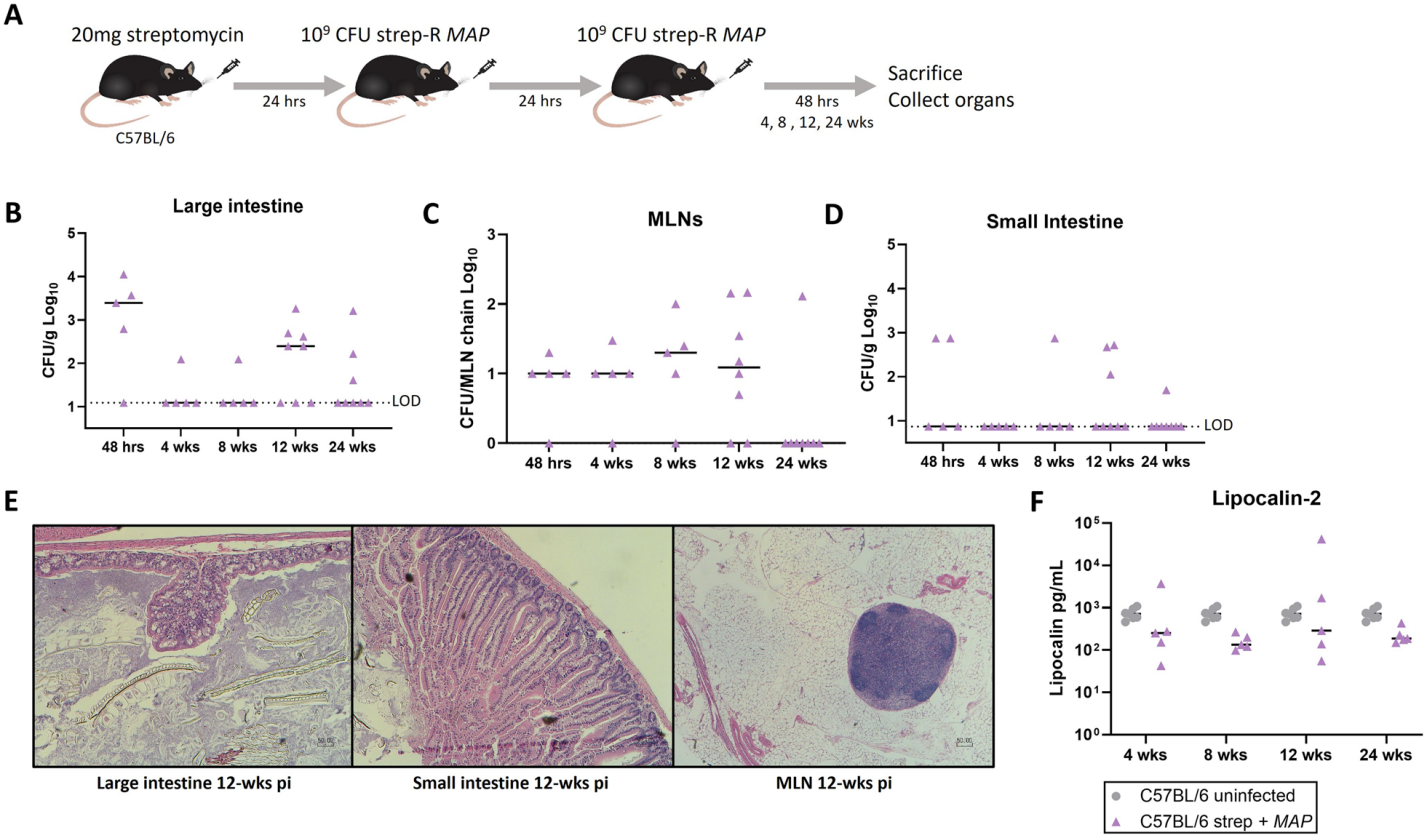
Assessment of chronic infection and disease following streptomycin pre-treatment and *MAP *gavage. **A** Mice were pre-treated with 20 mg streptomycin followed by 2 consecutive doses of 10^9^ CFU strep-R *MAP* given 24-hours apart. Mice were euthanized and assessed at 48-hours, 4-, 8-, 12-, and 24-weeks post-infection. **B**–**D** Strep-R *MAP* CFUs were assessed in the large intestine (**B**), MLNs (**C**), and small intestine (**D**) throughout the 24-week experiment. **E** Representative sections of the large intestine (H&E, original magnification x100), small intestine (x100), and MLNs (x40) from mice 12-weeks post-*MAP* infection are shown. **F** Fecal pellets were obtained from uninfected mice and strep-R *MAP* infected mice 4-, 8-, 12-, and 24-weeks post-infection and processed. The amount of lipocalin-2 found in samples from both groups was evaluated by ELISA

To determine whether the streptomycin pre-treatment *MAP* model led to long-term infection, the inoculation was repeated with timepoints extending to 24-weeks post-infection (Fig. 3A). Strep-R *MAP* was found to persist primarily in the large intestine (Fig. 3B) and MLNs (Fig. 3C) with CFUs sporadically observed in the small intestine (Fig. 3D). At initial uptake, *MAP* infects the large intestine (~ 10^3^-10^4^ CFU/g) and MLNs (~ 10^1^ CFU/MLN chain). At 4- and 8-weeks post-infection *MAP* was primarily detected in the MLNs (~ 10^1^-10^2^ CFU/MLN chain). By 12-weeks post-infection *MAP* was detected at greatest abundance in the large intestine (~ 10^2^-10^3^ CFU/g). At 24-weeks post-infection, *MAP* infection remained in some mice but had been cleared in most. *MAP* was not consistently detected in the spleen, liver, or lungs throughout the 24-week experiment (Additional file 1: Fig. S2). Considering 12-weeks post-gavage was the peak of *MAP* infection in the large intestine, fecal shedding was assessed via quantitative PCR of the F57 gene between uninfected controls and mice infected 12 weeks prior. No observable differences were detected between these 2 groups at this timepoint and the ge values were consistent with background (Additional file 1: Fig. S3).

To determine whether chronic infection led to inflammation or observable differences in histopathology, organs were sent for processing and H&E staining. The slides of the large intestine, small intestine, and MLNs were compared with slides from controls at the 12- and 24-week timepoints by a pathologist. These timepoints were chosen since *MAP* disease progression typically occurs after a long subclinical period and the 12-week timepoint was the peak of bacterial load in the large intestine. No changes were observed in the histopathology of infected animal organs compared to uninfected controls (Fig. 3E). To investigate whether more subtle inflammatory changes had occurred, fecal pellets were also processed for quantification of lipocalin-2, a broad marker for inflammation in mice [37] (Fig. 3F). No differences were observed in levels of lipocalin-2 compared to uninfected controls. Together this data suggests that a streptomycin pre-treatment model can result in long-term (up to 24 weeks) organ infection of *MAP* in C57BL/6 mice without signs of inflammation or disease.

### The infection outcomes of a streptomycin pre-treatment *MAP* model are comparable between C57BL/6 and BALB/c mice

**Fig. 4:**
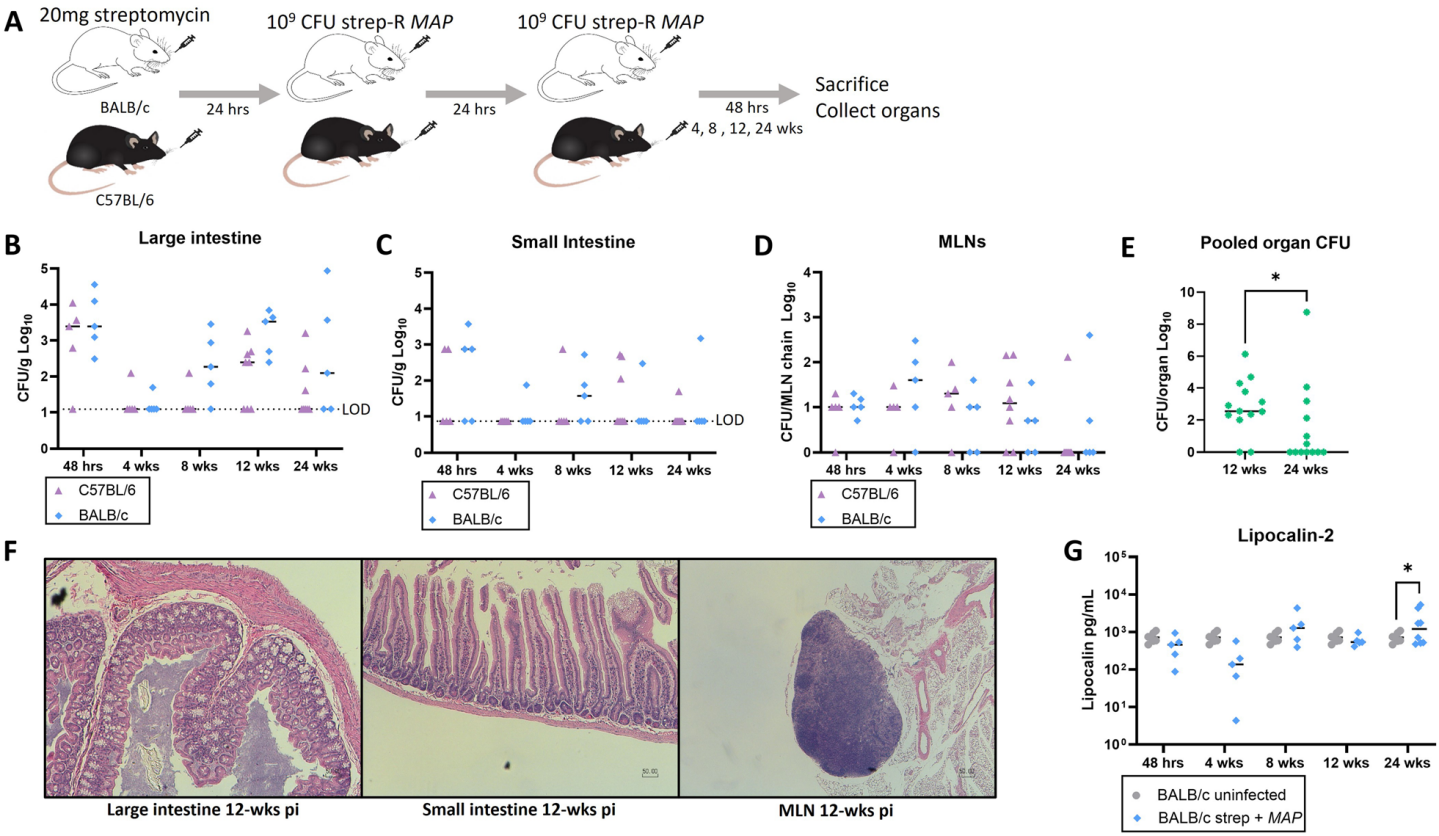
Comparison of infection outcomes between C57BL/6 and BALB/c mice. To evaluate whether using a more susceptible mouse strain would induce disease following strep-R *MAP* infection, the infection model was compared with BALB/c mice. **A** Either C57BL/6 or BALB/c mice were given 20 mg streptomycin followed by 2 consecutive doses of 10^9^ CFU strep-R *MAP*. Mice were sacrificed and assessed at 48-hours, 4-, 8-, 12-, and 24-weeks post-infection. **B**-**D** Strep-R *MAP* CFUs were compared between the 2 mice strains in the large intestine (**B**), small intestine (**C**), and MLNs (**D**). **E** Total *MAP* burden was pooled for the large intestine, small intestine, and MLNs of C57BL/6 and BALB/c mice and compared at 12- and 24-weeks post-infection (*p < 0.05) **F** Representative sections of the large intestine (H&E, original magnification x100), small intestine (x100), and MLNs (x40) from BALB/c mice 12-weeks post-MAP infection are shown. **G**. Fecal pellets were obtained from uninfected and strep-R *MAP* infected BALB/c mice 4-, 8-, 12-, and 24-weeks post-infection and processed. The amount of lipocalin-2 found in samples from both groups were evaluated by ELISA (*p < 0.05)

To identify whether infecting a mouse strain more susceptible to mycobacteria [39] would lead to increased bacterial burden or disease induction, the model was compared between C57BL/6 and BALB/c mice (Fig. 4A). Overall, no significant differences were observed between the 2 mouse strains in strep-R *MAP* infection of the large intestine (Fig. 4B), small intestine (Fig. 4C) or MLNs (Fig. 4D). This suggests that *MAP* organ infection is not affected by the differences between the genotypes of the C57BL/6 and BALB/c mice. Based on the comparable results across the different mouse strains, we pooled the results together to explore any time-dependent trends. Analysis of total CFU burden revealed that infection peaked at 12-weeks post-gavage and that the burden was diminished by 24-weeks (*p = 0.045) (Fig. 4E). This trend was seen also by analysis of each organ with a significant difference observed in the MLNs (*p = 0.049) (Additional file 1: Fig. S4). Consistent with the findings from C57BL/6 mice, no changes were observed in the histopathology of BALB/c mice compared to unexposed controls (Fig. 4F). The levels of lipocalin-2 were also comparable between the 2 groups, other than at 24-weeks where a minor increase was observed in infected animals (*p = 0.042) (Fig. 4G). When streptomycin pre-treatment was compared with no pre-treatment in BALB/c mice, strep-R *MAP* CFUs were shown to increase in the large intestine and MLNs with streptomycin pre-treatment. Slight increases in lipocalin-2 were observed in the streptomycin group compared to no pre-treatment (Additional file 1: Fig. S5). Overall, this data suggests that infection outcomes are largely unchanged by using BALB/c mice.

### The streptomycin pre-treatment infection strategy with *MAH* leads to comparatively transient infection

**Fig. 5:**
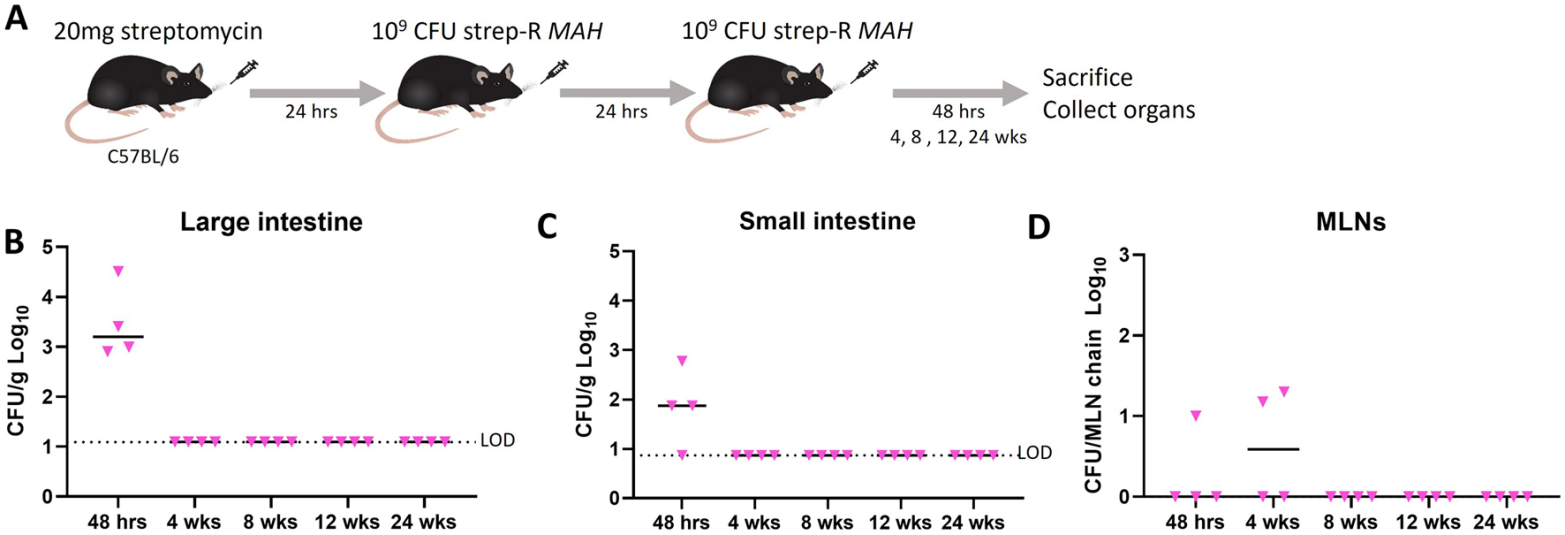
Comparison of the infection model with *MAH*. To determine how infection outcomes would compare using an environmental mycobacterium, the same infection strategy was repeated using strep-R *MAH*. **A** C57BL/6 mice were given 20 mg of streptomycin followed by 2 consecutive doses of strep-R *MAH* 24-hours apart. Mice were euthanized 48-hours, 4-, 8-, 12-, and 24-weeks post-infection and organs were assessed for CFUs. **B**–**D** Strep-R *MAH* CFUs were assessed in the large intestine (**B**), small intestine (**C**), and MLNs (**D**) at each timepoint

To understand how infection outcomes compare when the same infection strategy was applied to an environmental mycobacterium, the infection was repeated using *MAH*, the closest relative to *MAP* [8]. A strep-R strain of *MAH* was generated using oligo-mediated recombineering to introduce a K43R mutation in the *rpsL* gene and was confirmed by Sanger sequencing (Additional file 1: Fig. S6A). C57BL/6 mice were given 20 mg streptomycin followed by 2 consecutive doses of 1 × 10^9^ CFU strep-R *MAH* (Fig. 5A). Organ CFUs were found in the large and small intestine only 48-hours post-infection, after which strep-R *MAH* was cleared (Fig. 5B, C). Bacterial load ranged from ~ 10^3^-10^4^ CFUs/g in the large intestine, which is comparable to *MAP* CFUs at 48-hours (Fig. 3B). In the MLNs, infection was observed in some mice at 48-hours and 4-weeks post-infection, after which it was also cleared (Fig. 5D). Collectively, this data suggests that the persistence phenotype of *MAP* observed following this infection strategy is specific to the bacteria rather than the mode of infection.

### Application of the streptomycin pre-treatment strategy with *M. bovis* or *M. orygis* leads to pulmonary infection and disease

**Fig. 6:**
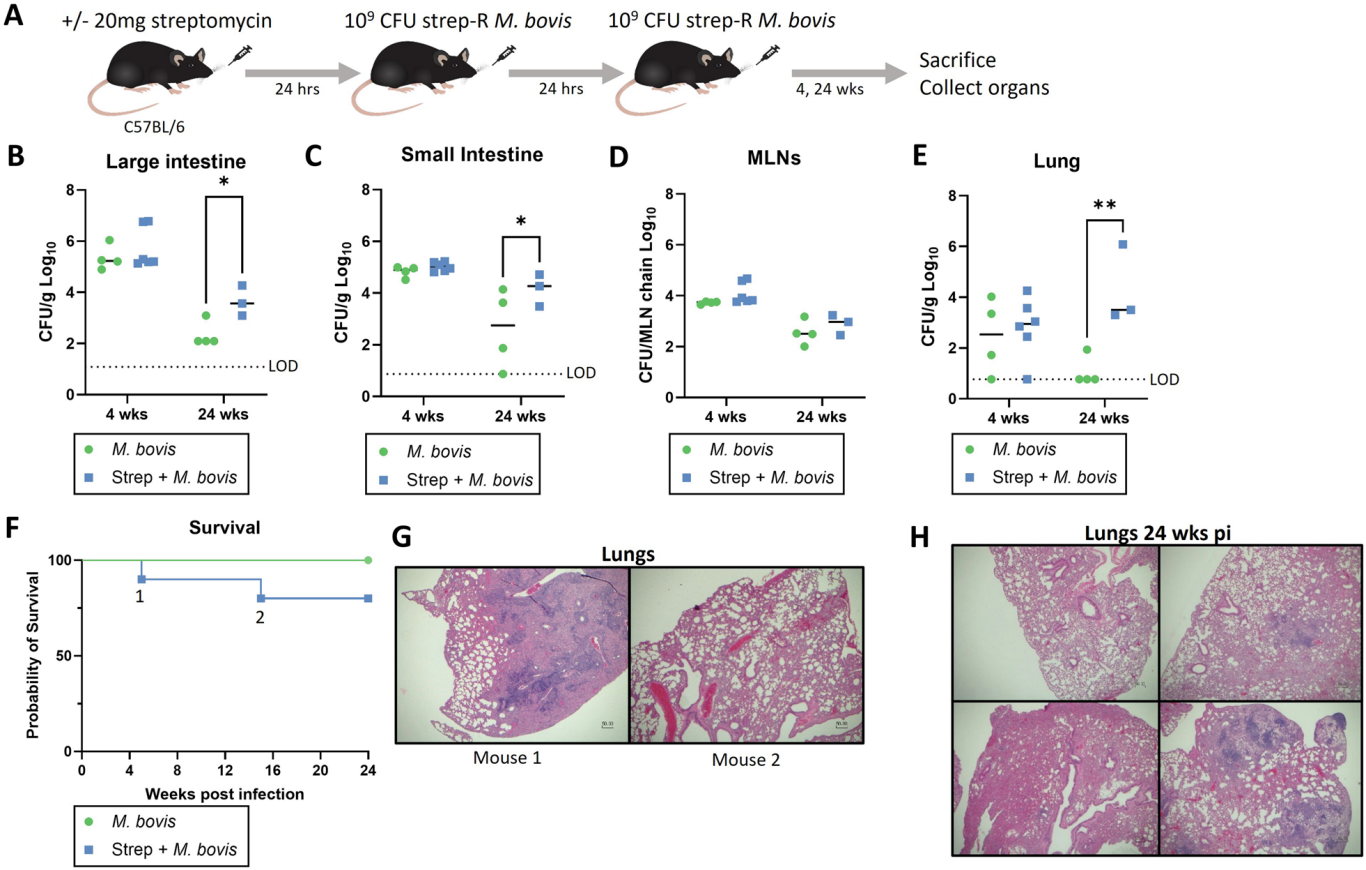
Comparison of the infection model with *M. bovis*. To determine how infection outcomes may change using a pathogenic tuberculous mycobacterium, the same infection strategy was repeated using strep-R *M. bovis*. **A** C57BL/6 mice were given 20 mg of streptomycin or no pre-treatment followed by 2 consecutive doses of strep-R *M. bovis* 24-hours apart. Mice were sacrificed 4- and 24-weeks post-infection and organs were assessed for CFUs. **B**–**D** Strep-R *M. bovis* CFUs were assessed between mice given streptomycin pre-treatment or no pre-treatment in the large intestine (**B**), small intestine (**C**), MLNs (**D**) and lungs (**E**) at each timepoint (*p < 0.05, **p < 0.01). **F** Survival was monitored between groups over the 24-week experiment. Two mice were flagged for euthanasia at 5-weeks (mouse 1) and 15-weeks (mouse 2) post-infection. **G** The histopathology of the lungs was assessed for the 2 mice that reached endpoint. Shown are representative images of sections of their lungs (H&E, original magnification x40). **H** At the experimental endpoint of 24-weeks, the histopathology of the lungs was assessed for the surviving mice. Representative images of the lungs sections (x40) from streptomycin pre-treated mice are pictured

To investigate whether the streptomycin pre-treatment infection model could lead to disease with more pathogenic species, infection outcomes were compared with *M. bovis* and *M. orygis*, 2 bovine mycobacteria causing tuberculosis. Strep-R *M. bovis/M. orygis* strains were generated by oligo-mediated recombineering and the K43R mutation was confirmed by Sanger sequencing (Additional file 1: Fig. S6B). For *M. bovis* infections, C57BL/6 mice were given 20 mg streptomycin or no pre-treatment followed by 2 consecutive oral doses of 1 × 10^9^ CFU of strep-R *M. bovis* each spaced 24-hours apart (Fig. 6A). Strep-R *M. bovis* infection consistently occurred in the large intestine (Fig. 6B), small intestine (Fig. 6C), and MLNs (Fig. 6D). Dissemination was also detected in the lungs (Fig. 6E). At 24-weeks post-infection, streptomycin pre-treatment led to significantly increased CFUs in the large intestine (*p = 0.041), small intestine (*p = 0.048) and lungs (**p = 0.0085). Over the course of the experiment, 2 mice in the streptomycin pre-treatment group had to be euthanized at 5-weeks (mouse 1) and 15-weeks (mouse 2) post-infection (Fig. 6F). Assessment of the histopathology of their lungs indicated that mouse 1 likely died due to lung disease due to the large areas of consolidation and immune cell infiltration observed. The lungs of mouse 2 appeared normal which indicated it may have died due to reasons unrelated to *M. bovis* infection (Fig. 6G). At the experimental endpoint of 24-weeks, sections of the lungs of the remaining mice were also sent for histopathology which indicated that 3 out of 4 mice had some evidence of lung disease including airway consolidation and interstitial lymphocytic infiltration (Fig. 6H). None of the *M. bovis-*infected mice had evidence of disease in their small or large intestine.

To determine whether similar infection outcomes would occur with another cause of bovine and zoonotic TB, the infection was repeated with strep-R *M. orygis* (Additional file 1: Fig. S7A). Similar outcomes were observed in this experiment where gavage led to local infection of the large intestine, small intestine, and MLNs with dissemination to the lungs at 24-weeks post-infection (Additional file 1: Fig. S7B–E). One mouse died at 13-weeks post-infection without evidence of lung disease observed by H&E staining (Additional file 1: Fig. S7F, G). The surviving mice that were euthanized at the experimental endpoint had some evidence of lung disease with areas showing reactive peri-bronchial lymphoid aggregates, interstitial lymphocytic infiltration and foamy histiocytes (Additional file 1: Fig. S7H). None of the *M. orygis-*infected mice had evidence of disease in their small or large intestine.

## Discussion

Paratuberculosis remains a widespread concern for animal health and a significant economic burden. The limitations of available diagnostics and vaccines for paratuberculosis deters elimination of the disease. The development of a mouse model of *MAP* which mimics the natural route of infection and leads to intestinal disease would be a valuable tool for testing new methods of detecting or preventing paratuberculosis. However, oral infection models of *MAP* are currently lacking. Here, we investigated whether streptomycin pre-treatment, a method previously shown to support *S. enterica* serovar Typhimurium induced enteritis, could improve *MAP* infections and model paratuberculosis in mice.

Streptomycin pre-treatment was found to significantly improve *MAP* infection of the large intestine (Fig. 1B). The benefit of streptomycin pre-treatment to increase organ bacterial load was also found when infecting another mouse strain (BALB/c) or when inoculating another mycobacterium (*M. bovis*) (Additional file 1: Figs. S5 and S6). To further enhance *MAP* infection, the dosage was increased to 2 consecutive doses of 10^9^ and only mice that received this higher dose were shown to have *MAP* infection in the MLNs (Fig. 2). During the clinical phase of paratuberculosis, inflamed MLNs are one of the frequently observed signs of disease [11] thus this higher dosage led to a more representative *MAP* model. Streptomycin pre-treatment followed by a high dose *MAP* gavage led to a persistent *MAP* infection in the intestines and MLNs which peaked at 12-weeks. Our strategy for assessing intestinal CFUs was designed to detect bacterial burden within the organ tissue. It is possible that additional CFUs may have been detected within the intestines if the mucus was not washed away during processing. Although the improvement in *MAP* burden using this streptomycin pre-treatment was promising, there was no evidence of inflammation or disease in either C57BL/6 or BALB/c mice (Figs. 3 and 4). Furthermore, there was no evidence that *MAP* was being shed through the feces at the time of the maximum infection burden. Future studies attempting to model paratuberculosis in mice may benefit from a streptomycin pre-treatment strategy to enhance *MAP* infection. However, given that disease was not generated at an inoculum that is difficult to exceed, for technical reasons, additional host manipulations such as an immunodeficient mouse strain are likely required to generate *MAP* enteritis.

In addition to streptomycin pre-treatment, our study investigated the effect of gastric pH on infection outcomes. Although in vitro studies showed that a low pH like the environment of the mouse stomach did lead to reduced *MAP* CFUs, when sodium bicarbonate was used to increase the mouse gastric pH prior to gavage with *MAP*, no differences were observed in the organ CFUs compared to mice given no sodium bicarbonate (Fig. 1C–G). It is possible that the effect seen in vitro did not translate into a detectable difference in vivo, simply because of the amount of time *MAP* spends in the mouse stomach. Schwartz *et al. *reported the mouse gastric emptying time to be 74 ± 17 min but also reported considerable variability between animals [40]. Given that the tissue burden of *MAP* was much lower than the inoculum we delivered via gavage, our results suggest that there are other pH-independent factors which hinder *MAP* infection post-oral inoculation.

To determine how infection outcomes may vary when using the same infection strategy with mycobacteria of lesser or greater virulence, the infection was compared with *MAH*, a primarily environmental bacteria, and *M. bovis/M. orygis*, bacteria known to cause bovine tuberculosis. Gavage of *MAH* led to a transient infection in the large intestine and MLNs which was cleared after 48-hours or 4-weeks post-infection respectively (Fig. 5). This indicated that the persistence of *MAP* was specific to the bacteria rather than the method of streptomycin pre-treatment. When mice were inoculated with *M. bovis* or *M. orygis*, this led to both infection of the intestines and MLNs, and dissemination to the lungs. After 24 weeks, these mice had clear signs of lung disease which varied in severity. Overall, these infection outcomes were consistent with a model of zoonotic tuberculosis where exposure via the oral route led to pulmonary disease. This indicates that *MAP* localization and lack of disease development within the streptomycin pre-treatment model was again bacteria-specific rather than a product of the infection strategy.

In conclusion, our data suggests that streptomycin pre-treatment is an effective method for improving infection of *MAP* post-gavage. When compared to *MAH, M. bovis*, and *M. orygis*, *MAP* gavage led to local persistent infection which was not observed with the other bacteria. Future studies aiming to develop an oral infection model of *MAP* may build upon these data to enhance infection and/or to produce an inflammatory response to *MAP* infection. A mouse model of paratuberculosis could act as an important platform for testing novel diagnostics and vaccines to improve management of paratuberculosis.

## Supplementary Information

**Additional file 1: Figure S1.** Generation of strep-R *MAP.* Oligo-mediated recombineering was employed to generate a K43R mutation in *rpsL* of *MAP* which is known to confer resistance to streptomycin. **A** The sequence of the *rpsL *gene of wildtype (WT)* MAP* and strep-R *MAP *was generated by Sanger sequencing. Amino acids 40-46 of* MAP rpsL* are visualized on Geneious Prime and the point mutation in strep-R *MAP *is highlighted by a green box. **B** The WT *MAP *and strep-R *MAP *strains were plated on 7H10 agar with streptomycin to compare growth. **Figure S2.** Dissemination of strep-R *MAP.* **A **C57BL/6 mice were given 20 mg of streptomycin followed by 2 consecutive doses of 10^9^ CFU strep-R *MAP* each 24-hours apart. **B**–**D** Dissemination of *MAP *into the spleen (**B**), liver (**C**), and lungs (**D**) was evaluated at 48-hours, 4-, 8-, 12-, and 24-weeks post-gavage. **Figure S3.** Fecal shedding assessment. **A** A standard curve was prepared for quantitative PCR of the F57 gene using *MAP* K10 genomic DNA diluted from 1x10^7^to 1 genome equivalents in order to interpolate values from fecal samples. **B** Fecal shedding was assessed in uninfected controls and mice 12-weeks post-gavage with *MAP*. **Figure S4.** Pooled organ CFUs of C57BL/6 and BALB/c mice at 12- and 24-weeks post-infection. The organ CFUs of the large intestine (**A**), small intestine (**B**), and MLNs (**C**) were pooled from C57BL/6 and BALB/c mice and compared between 12- and 24-weeks post-infection (*p<0.05). **Figure S5.** Comparison of streptomycin pre-treatment and no pre-treatment in BALB/c mice. To determine whether streptomycin pre-treatment would also increase infection in BALB/c mice, infection outcomes were compared between BALB/c mice given streptomycin pre-treatment or no pre-treatment. **A** BALB/c mice were given 20 mg streptomycin or no pre-treatment followed by 2 consecutive doses of 10^9^ CFU strep-R *MAP* each 24-hours apart. **B**–**D**. The CFUs of the large intestine (**B**), small intestine (**C**), and MLNs (**D**) were compared between mice groups 48-hours, 4-, 8-, 12-, and 24-weeks post-infection. **E** Fecal pellets were collected and processed from uninfected BALB/c mice and *MAP*-infected BALB/c mice with or without streptomycin pre-treatment. The levels of lipocalin-2 found in the feces of each group were evaluated by ELISA (*p < 0.05, **p < 0.01). **Figure S6.** Generation of strep-R *MAH *and strep-R *M. bovis*. Oligo-mediated recombineering was employed to generate a K43R mutation in *rpsL *of *MAH *and *M. bovis. ***A** The sequence of the *rpsL* gene of WT *MAH* and strep-R *MAH *was generated by Sanger sequencing. Amino acids 42-44 of *MAH rpsL* were visualized on Geneious Prime and the point mutation in strep-R *MAH *is indicated by a green box. **B** The sequence of the *rpsL* gene of WT *M. bovis* and strep-R *M. bovis* was generated by Sanger sequencing. Amino acids 42-44 of *M. bovis rpsL* were visualized on Geneious Prime and the point mutation in strep-R *M. bovis* is highlighted by a green box. **Figure S7**. Comparison of infection model with *M. orygis.* The streptomycin pre-treatment model was repeated with another pathogenic bovine mycobacterium *M. orygis.* **A** C57BL/6 mice were given 20 mg of streptomycin followed by 2 consecutive doses of strep-R *M. orygis *24-hours apart. Mice were euthanized 4- and 24-weeks post-infection. **B**–**D**. Strep-R *M. orygis* CFUs were assessed in the large intestine (**B**), small intestine (**C**), MLNs (**D**) and lungs (**E**) at each timepoint. **F** Survival was monitored over the 24-week experiment. One mouse was flagged for euthanasia at 13-weeks post-infection (mouse 1). **G** The histopathology of the lungs was assessed for mouse 1. Shown is a representative image of the H&E-stained section of its lungs (original magnification x40). **H** At the experimental endpoint of 24-weeks, the histopathology of the lungs was assessed for the surviving mice. Shown are representative images of the lungs (x40) sections. **Table S1.** Primer and oligo sequences.

## Data Availability

All data generated or analyzed during this study are included in this published article and its supplementary information.

## Abbreviations

CCAC: Canadian council on animal care
CFU: Colony-forming units
GE: Genome equivalents
H&E: Hematoxylin and eosin
LOD: Limit of detection
*MAH.*: *Mycobacterium avium* subsp. *hominissuis*
*MAP *: *Mycobacterium avium* subsp. *paratuberculosis*
MLN: Mesenteric lymph node
RI-MUHC: Research Institute of the McGill University Health Centre
Strep-R: Streptomycin-resistant
WT: Wildtype
