# Correction: Establishment of persistent enteric mycobacterial infection following streptomycin pre-treatment

**DOI:** 10.1186/s13099-024-00649-1

**Published:** 2024-09-29

**Authors:** Shannon C. Duffy, Andréanne Lupien, Youssef Elhaji, Mina Farag, Victoria Marcus, Marcel A. Behr

**Affiliations:** 1 Department of Microbiology and Immunology, McGill University, Montreal, QC Canada; 2 McGill International TB Centre, Montreal, QC Canada; 3 The Infectious Diseases and Immunity in Global Health Program, The Research Institute of the McGill University Health Centre, Montreal, QC Canada; 4 Department of Medicine, McGill University, Montreal, QC Canada; 5 Diagnostic Genomic Division, Department of Laboratory Medicine and Pathology, Hamad Medical Corporation, Doha, Qatar; 6 Department of Pathology, McGill University, Montreal, QC Canada; 7 Department of Laboratory Medicine, Division of Pathology, McGill University Health Center, Montreal, QC Canada

**Correction to: Gut Pathogens (2023) 15:46**

10.1186/s13099-023-00573-w

In this article [1], the wrong figure appeared as Figs. 3F and 4G and Supp. Figure 5E. The corrected Figs. 3 and 4 and the corrected supplement Fig. 5 are given in this correction.

**Fig. 3:**
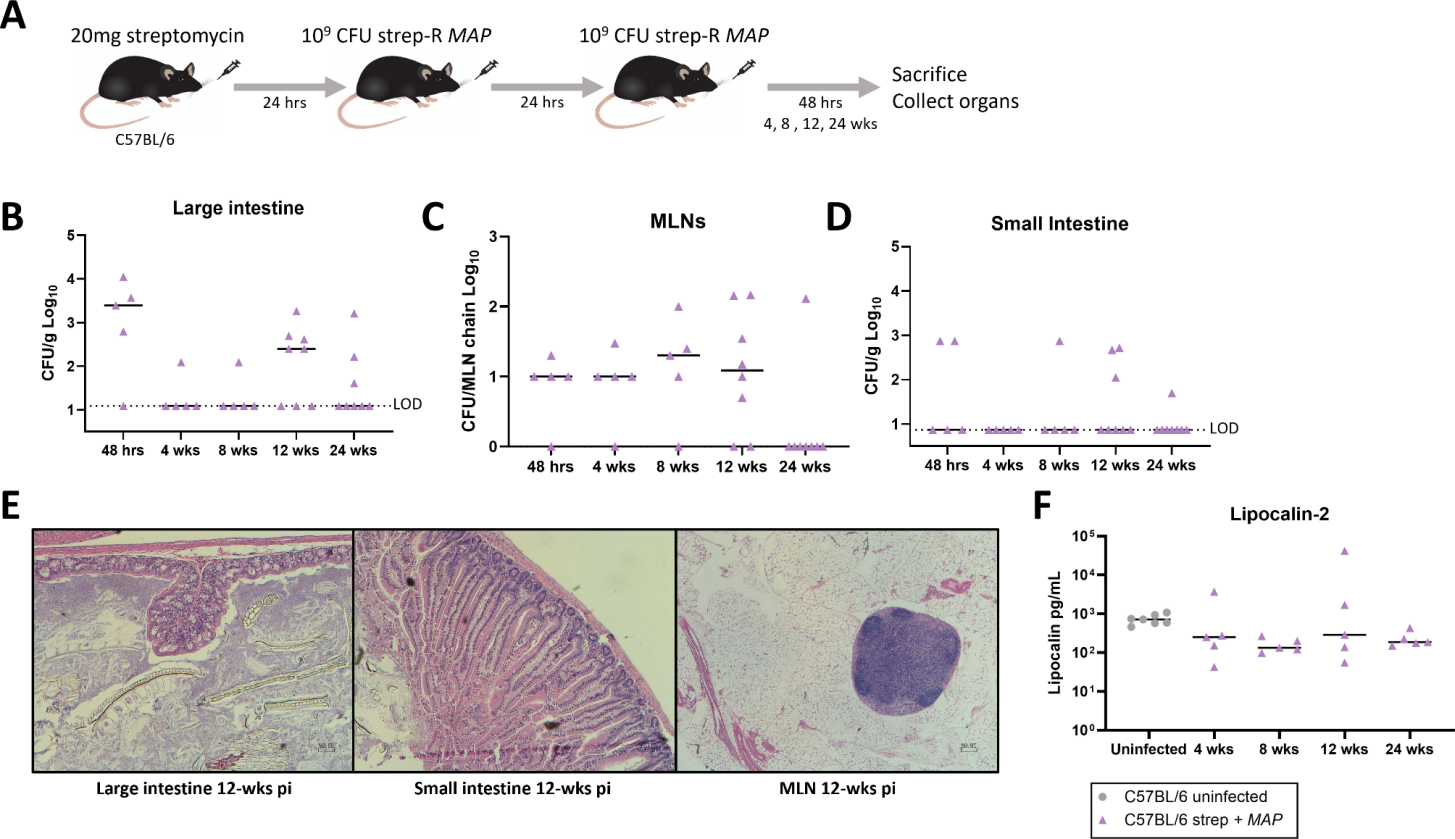
Assessment of chronic infection and disease following streptomycin pre-treatment and *MAP* gavage. **A** Mice were pre-treated with 20 mg streptomycin followed by 2 consecutive doses of 109 CFU strep-R *MAP* given 24-hours apart. Mice were euthanized and assessed at 48-hours, 4-, 8-, 12-, and 24-weeks post-infection. **B**–**D** Strep-R *MAP* CFUs were assessed in the large intestine (**B**), MLNs (**C**), and small intestine (**D**) throughout the 24-week experiment. **E** Representative sections of the large intestine (H&E, original magnification x100), small intestine (x100), and MLNs (x40) from mice 12-weeks post-*MAP* infection are shown. **F** Fecal pellets were obtained from uninfected mice and strep-R *MAP* infected mice 4-, 8-, 12-, and 24-weeks post-infection and processed. The amount of lipocalin-2 found in samples from both groups was evaluated by ELISA

**Fig. 4:**
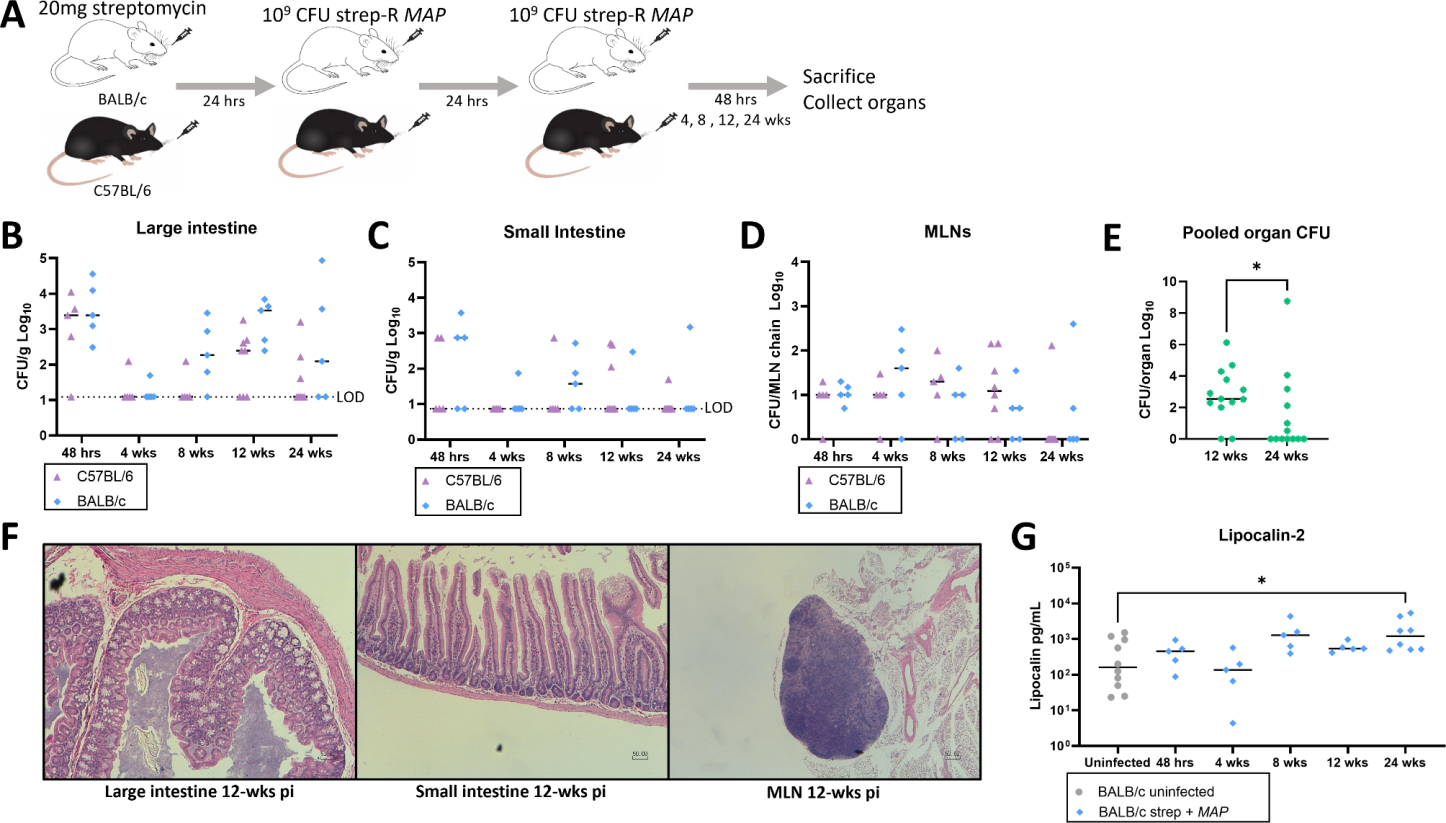
Comparison of infection outcomes between C57BL/6 and BALB/c mice. To evaluate whether using a more susceptible mouse strain would induce disease following strep-R *MAP* infection, the infection model was compared with BALB/c mice. **A** Either C57BL/6 or BALB/c mice were given 20 mg streptomycin followed by 2 consecutive doses of 109 CFU strep-R *MAP*. Mice were sacrificed and assessed at 48-hours, 4-, 8-, 12-, and 24-weeks post-infection. **B**-**D** Strep-R *MAP* CFUs were compared between the 2 mice strains in the large intestine (**B**), small intestine (**C**), and MLNs (**D**). **E** Total *MAP* burden was pooled for the large intestine, small intestine, and MLNs of C57BL/6 and BALB/c mice and compared at 12- and 24-weeks post-infection (*p < 0.05). **F** Representative sections of the large intestine (H&E, original magnification x100), small intestine (x100), and MLNs (x40) from BALB/c mice 12-weeks post-MAP infection are shown. **G**. Fecal pellets were obtained from uninfected and strep-R *MAP* infected BALB/c mice 4-, 8-, 12-, and 24-weeks post-infection and processed. The amount of lipocalin-2 found in samples from both groups were evaluated by ELISA (*p < 0.05)

## Electronic supplementary material

Below is the link to the electronic supplementary material.

Supplementary Material 1
