# Supplementary material for: Establishment of persistent enteric mycobacterial infection following streptomycin pre-treatment
Source: Gut Pathog. 2023 Oct 3;15:46. doi: 10.1186/s13099-023-00573-w (PMC10546655; doi:10.1186/s13099-023-00573-w)
Supplement: Supplementary file 1 — Additional file 1: Figure S1. Generation of strep-R MAP. Oligo-mediated recombineering was employed to generate a K43R mutation in rpsL of MAP which is known to confer resistance to streptomycin. A The sequence of the rpsL gene of wildtype (WT) MAP and strep-R MAP was generated by Sanger sequencing. Amino acids 40-46 of MAP rpsL are visualized on Geneious Prime and the point mutation in strep-R MAP is highlighted by a green box. B The WT MAP and strep-R MAP strains were plated on 7H10 agar with streptomycin to compare growth. Figure S2. Dissemination of strep-R MAP. A C57BL/6 mice were given 20 mg of streptomycin followed by 2 consecutive doses of 109 CFU strep-R MAP each 24-hours apart. B–D Dissemination of MAP into the spleen (B), liver (C), and lungs (D) was evaluated at 48-hours, 4-, 8-, 12-, and 24-weeks post-gavage. Figure S3. Fecal shedding assessment. A A standard curve was prepared for quantitative PCR of the F57 gene using MAP K10 genomic DNA diluted from 1x10^7^to 1 genome equivalents in order to interpolate values from fecal samples. B Fecal shedding was assessed in uninfected controls and mice 12-weeks post-gavage with MAP. Figure S4. Pooled organ CFUs of C57BL/6 and BALB/c mice at 12- and 24-weeks post-infection. The organ CFUs of the large intestine (A), small intestine (B), and MLNs (C) were pooled from C57BL/6 and BALB/c mice and compared between 12- and 24-weeks post-infection (*p<0.05). Figure S5. Comparison of streptomycin pre-treatment and no pre-treatment in BALB/c mice. To determine whether streptomycin pre-treatment would also increase infection in BALB/c mice, infection outcomes were compared between BALB/c mice given streptomycin pre-treatment or no pre-treatment. A BALB/c mice were given 20 mg streptomycin or no pre-treatment followed by 2 consecutive doses of 109 CFU strep-R MAP each 24-hours apart. B–D. The CFUs of the large intestine (B), small intestine (C), and MLNs (D) were compared between mice groups 48-hours, 4-, 8-, 12-, and 24-wee [file 13099_2023_573_MOESM1_ESM.pptx]

## Slide 1
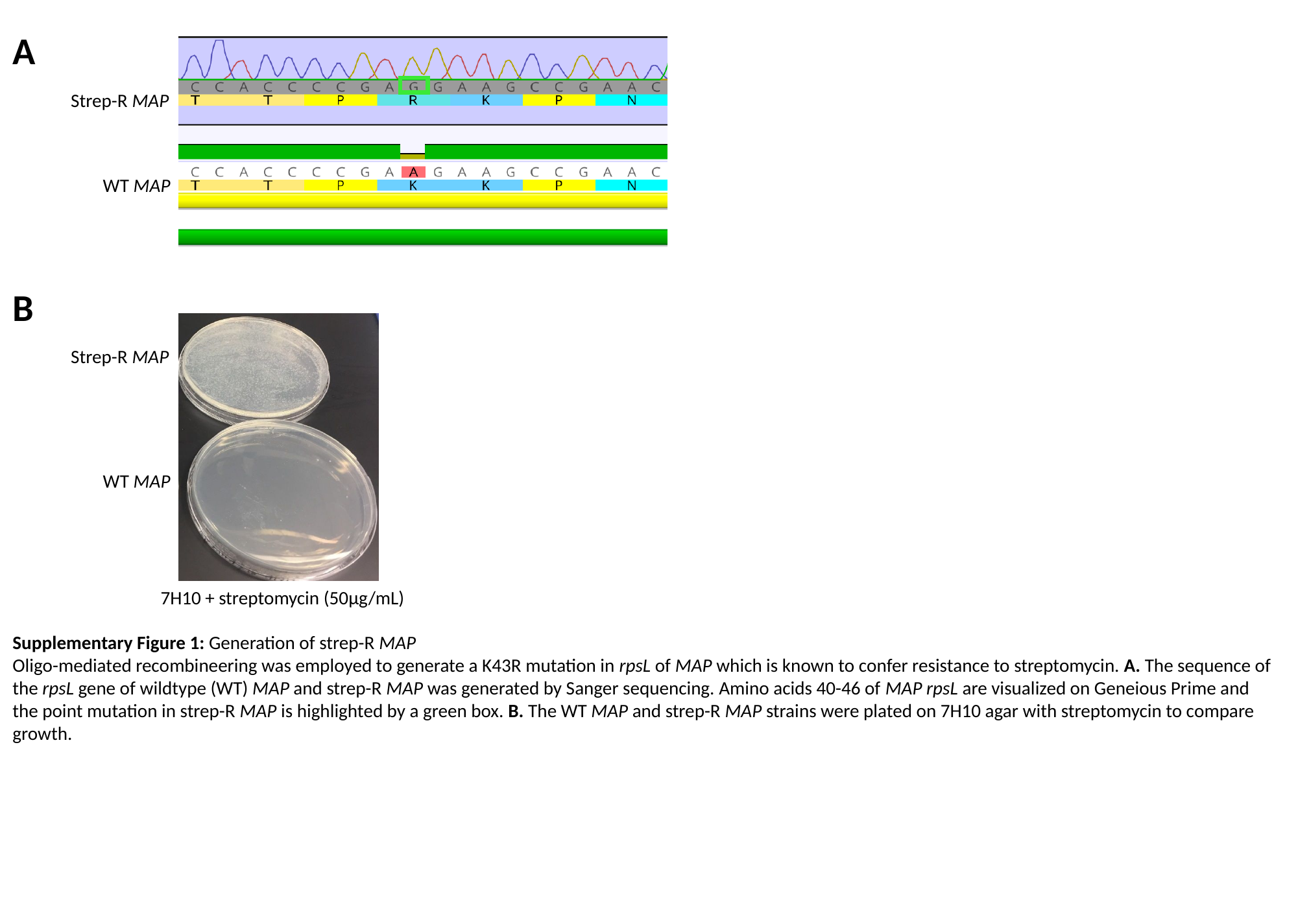

A
Strep-R MAP
WT MAP
B
Strep-R MAP
WT MAP
7H10 + streptomycin (50μg/mL)
Supplementary Figure 1: Generation of strep-R MAP
Oligo-mediated recombineering was employed to generate a K43R mutation in rpsL of MAP which is known to confer resistance to streptomycin. A. The sequence of the rpsL gene of wildtype (WT) MAP and strep-R MAP was generated by Sanger sequencing. Amino acids 40-46 of MAP rpsL are visualized on Geneious Prime and the point mutation in strep-R MAP is highlighted by a green box. B. The WT MAP and strep-R MAP strains were plated on 7H10 agar with streptomycin to compare growth.

## Slide 2
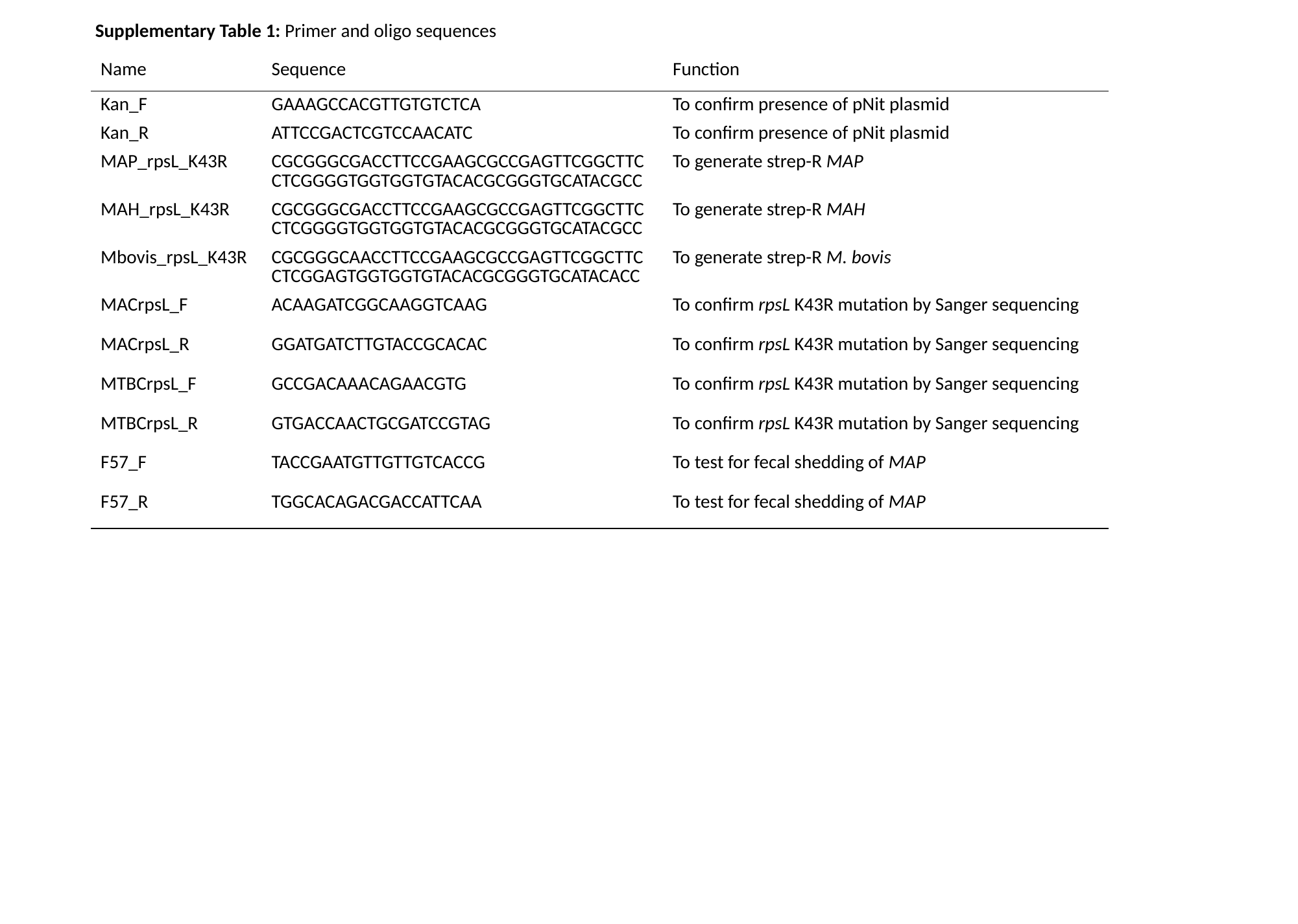

Supplementary Table 1: Primer and oligo sequences
| Name | Sequence | Function |
| --- | --- | --- |
| Kan\_F | GAAAGCCACGTTGTGTCTCA | To confirm presence of pNit plasmid |
| Kan\_R | ATTCCGACTCGTCCAACATC | To confirm presence of pNit plasmid |
| MAP\_rpsL\_K43R | CGCGGGCGACCTTCCGAAGCGCCGAGTTCGGCTTCCTCGGGGTGGTGGTGTACACGCGGGTGCATACGCC | To generate strep-R MAP |
| MAH\_rpsL\_K43R | CGCGGGCGACCTTCCGAAGCGCCGAGTTCGGCTTCCTCGGGGTGGTGGTGTACACGCGGGTGCATACGCC | To generate strep-R MAH |
| Mbovis\_rpsL\_K43R | CGCGGGCAACCTTCCGAAGCGCCGAGTTCGGCTTCCTCGGAGTGGTGGTGTACACGCGGGTGCATACACC | To generate strep-R M. bovis |
| MACrpsL\_F | ACAAGATCGGCAAGGTCAAG | To confirm rpsL K43R mutation by Sanger sequencing |
| MACrpsL\_R | GGATGATCTTGTACCGCACAC | To confirm rpsL K43R mutation by Sanger sequencing |
| MTBCrpsL\_F | GCCGACAAACAGAACGTG | To confirm rpsL K43R mutation by Sanger sequencing |
| MTBCrpsL\_R | GTGACCAACTGCGATCCGTAG | To confirm rpsL K43R mutation by Sanger sequencing |
| F57\_F | TACCGAATGTTGTTGTCACCG | To test for fecal shedding of MAP |
| F57\_R | TGGCACAGACGACCATTCAA | To test for fecal shedding of MAP |

## Slide 3
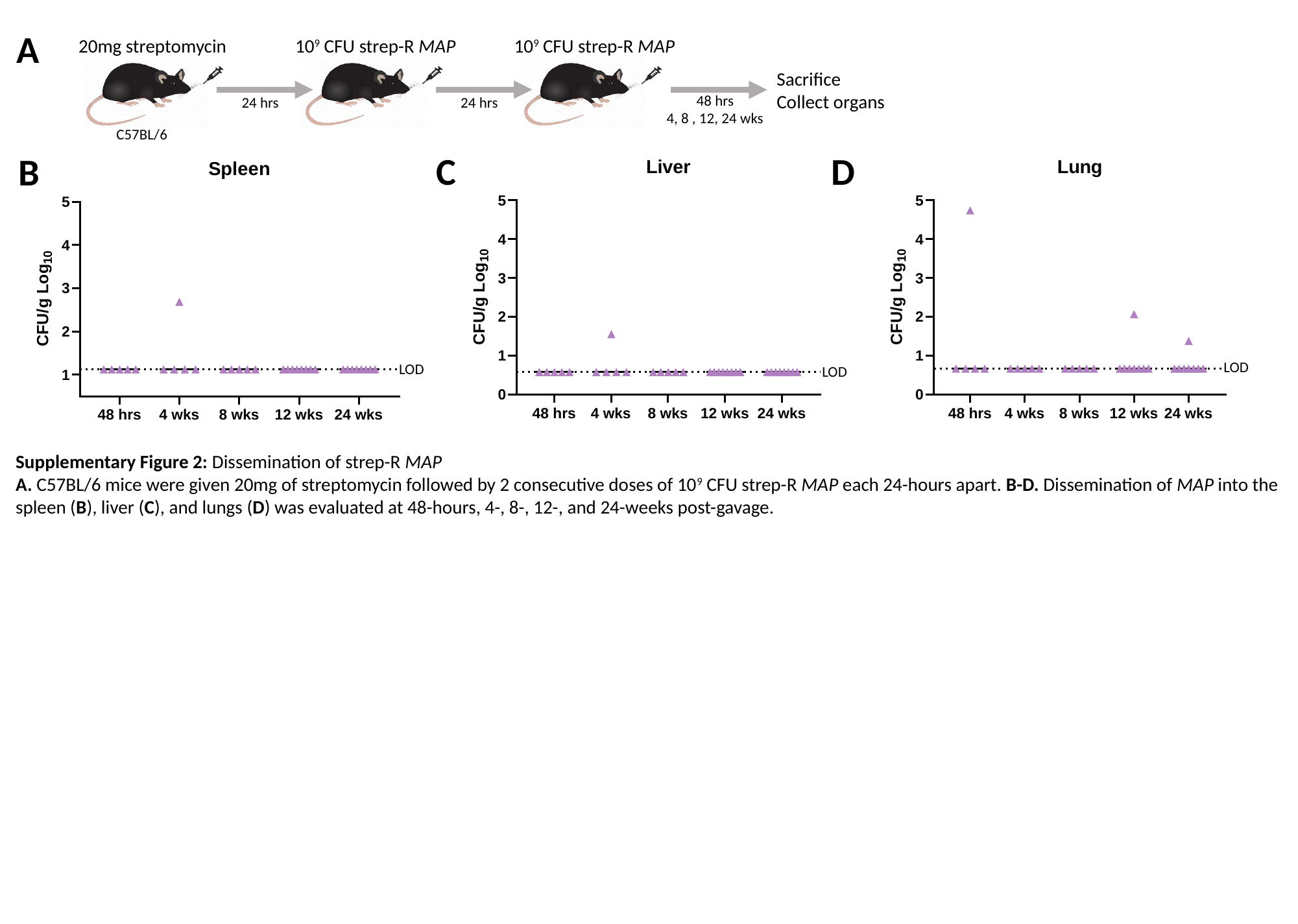

A
109 CFU strep-R MAP
20mg streptomycin
109 CFU strep-R MAP
Sacrifice
Collect organs
24 hrs
24 hrs
C57BL/6
48 hrs
4, 8 , 12, 24 wks
D
C
B
LOD
LOD
LOD
Supplementary Figure 2: Dissemination of strep-R MAP
A. C57BL/6 mice were given 20mg of streptomycin followed by 2 consecutive doses of 109 CFU strep-R MAP each 24-hours apart. B-D. Dissemination of MAP into the spleen (B), liver (C), and lungs (D) was evaluated at 48-hours, 4-, 8-, 12-, and 24-weeks post-gavage.

## Slide 4
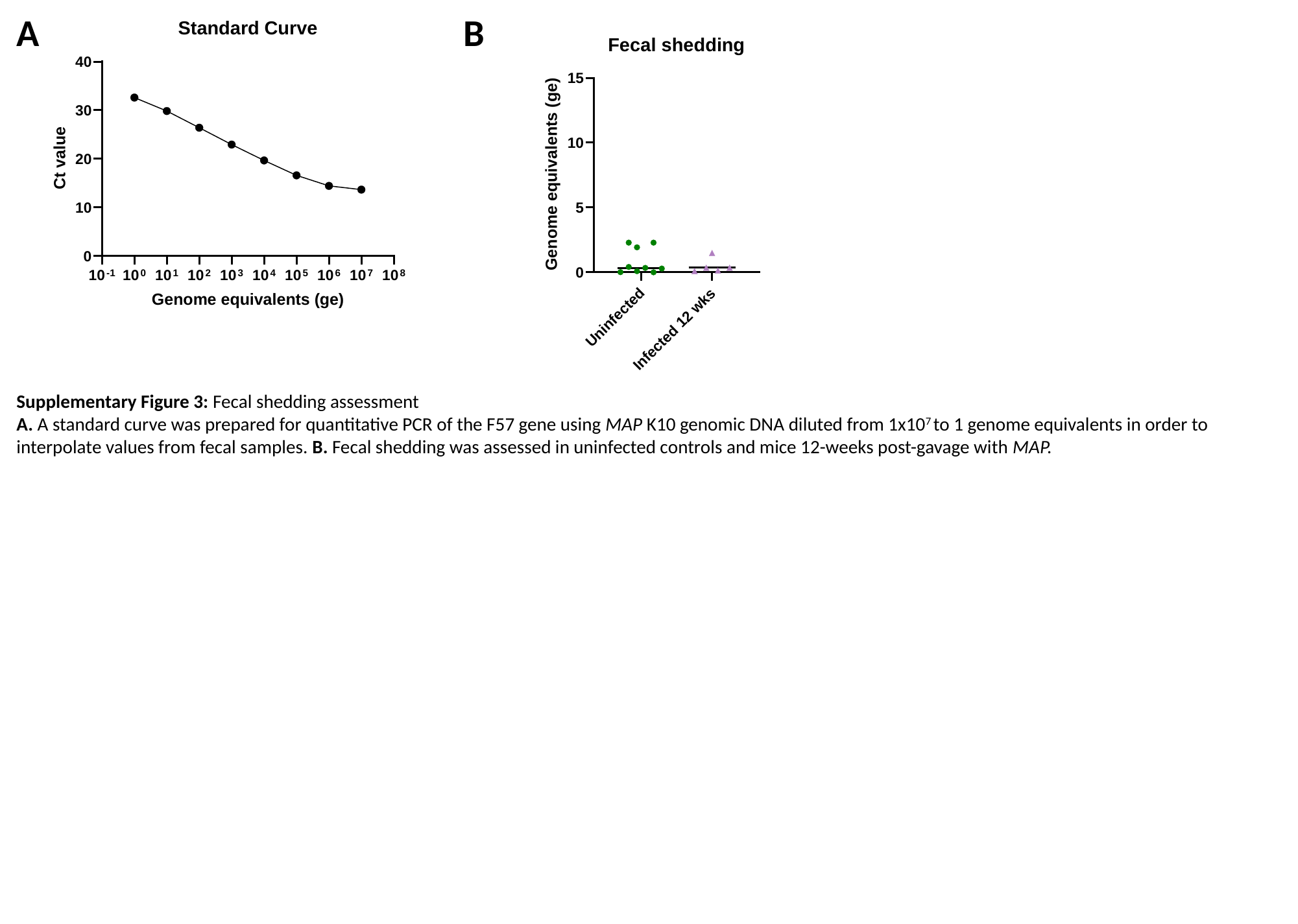

A
B
Supplementary Figure 3: Fecal shedding assessment
A. A standard curve was prepared for quantitative PCR of the F57 gene using MAP K10 genomic DNA diluted from 1x10^7^ to 1 genome equivalents in order to interpolate values from fecal samples. B. Fecal shedding was assessed in uninfected controls and mice 12-weeks post-gavage with MAP.

## Slide 5
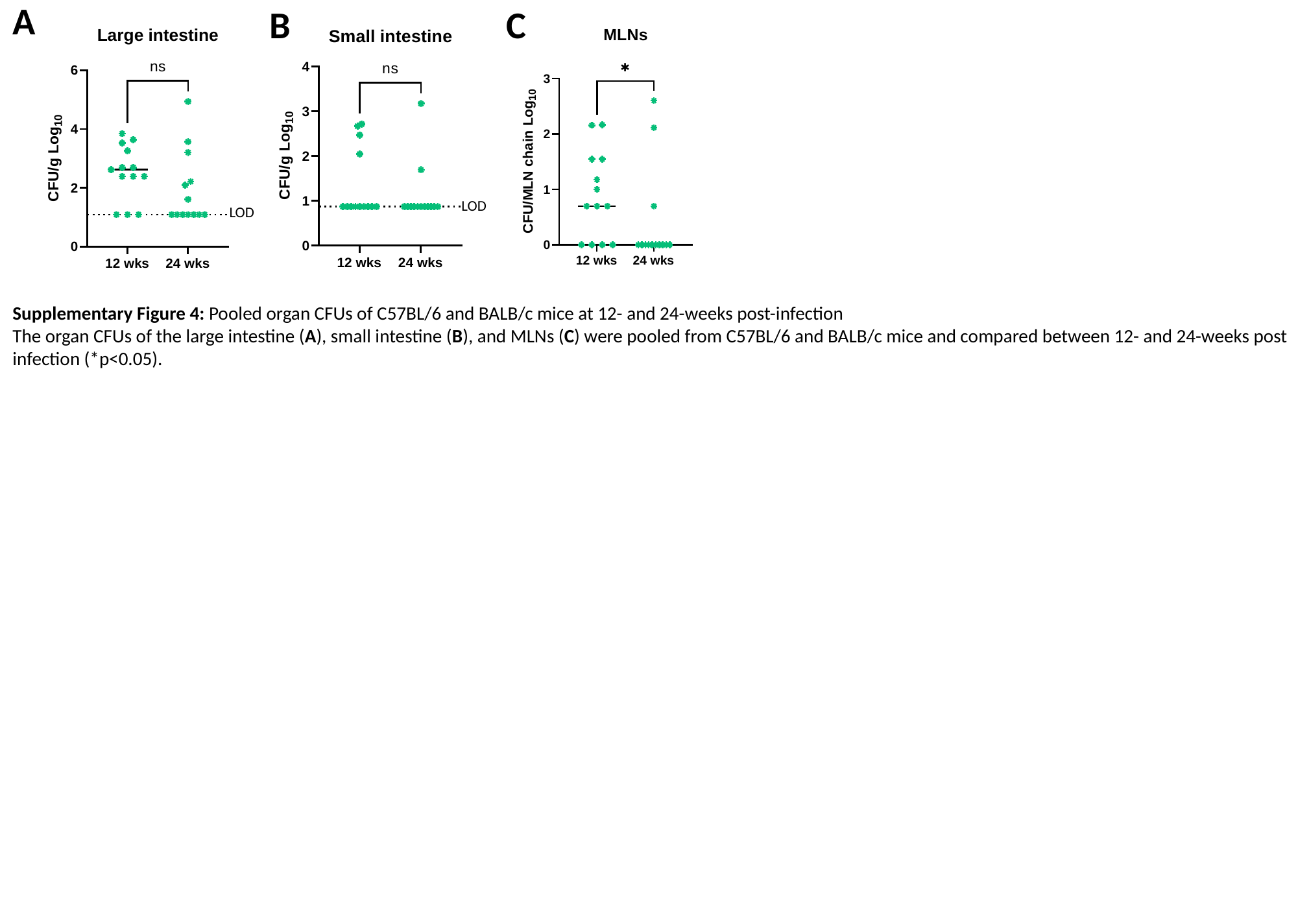

A
B
C
LOD
LOD
Supplementary Figure 4: Pooled organ CFUs of C57BL/6 and BALB/c mice at 12- and 24-weeks post-infection
The organ CFUs of the large intestine (A), small intestine (B), and MLNs (C) were pooled from C57BL/6 and BALB/c mice and compared between 12- and 24-weeks post infection (*p<0.05).

## Slide 6
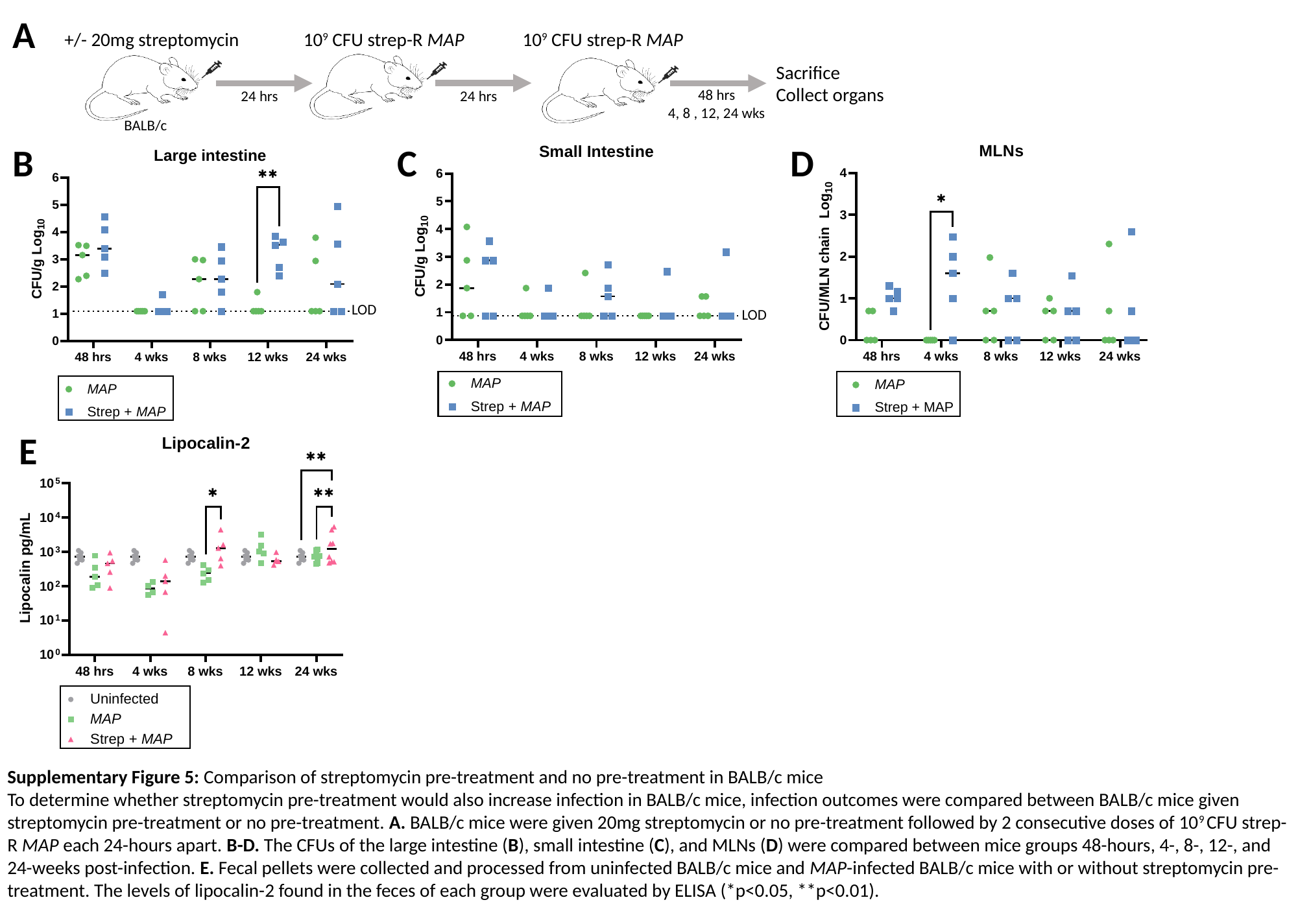

A
109 CFU strep-R MAP
+/- 20mg streptomycin
109 CFU strep-R MAP
Sacrifice
Collect organs
24 hrs
24 hrs
BALB/c
48 hrs
4, 8 , 12, 24 wks
B
C
D
LOD
LOD
E
Supplementary Figure 5: Comparison of streptomycin pre-treatment and no pre-treatment in BALB/c mice
To determine whether streptomycin pre-treatment would also increase infection in BALB/c mice, infection outcomes were compared between BALB/c mice given streptomycin pre-treatment or no pre-treatment. A. BALB/c mice were given 20mg streptomycin or no pre-treatment followed by 2 consecutive doses of 109 CFU strep-R MAP each 24-hours apart. B-D. The CFUs of the large intestine (B), small intestine (C), and MLNs (D) were compared between mice groups 48-hours, 4-, 8-, 12-, and 24-weeks post-infection. E. Fecal pellets were collected and processed from uninfected BALB/c mice and MAP-infected BALB/c mice with or without streptomycin pre-treatment. The levels of lipocalin-2 found in the feces of each group were evaluated by ELISA (*p<0.05, **p<0.01).

## Slide 7
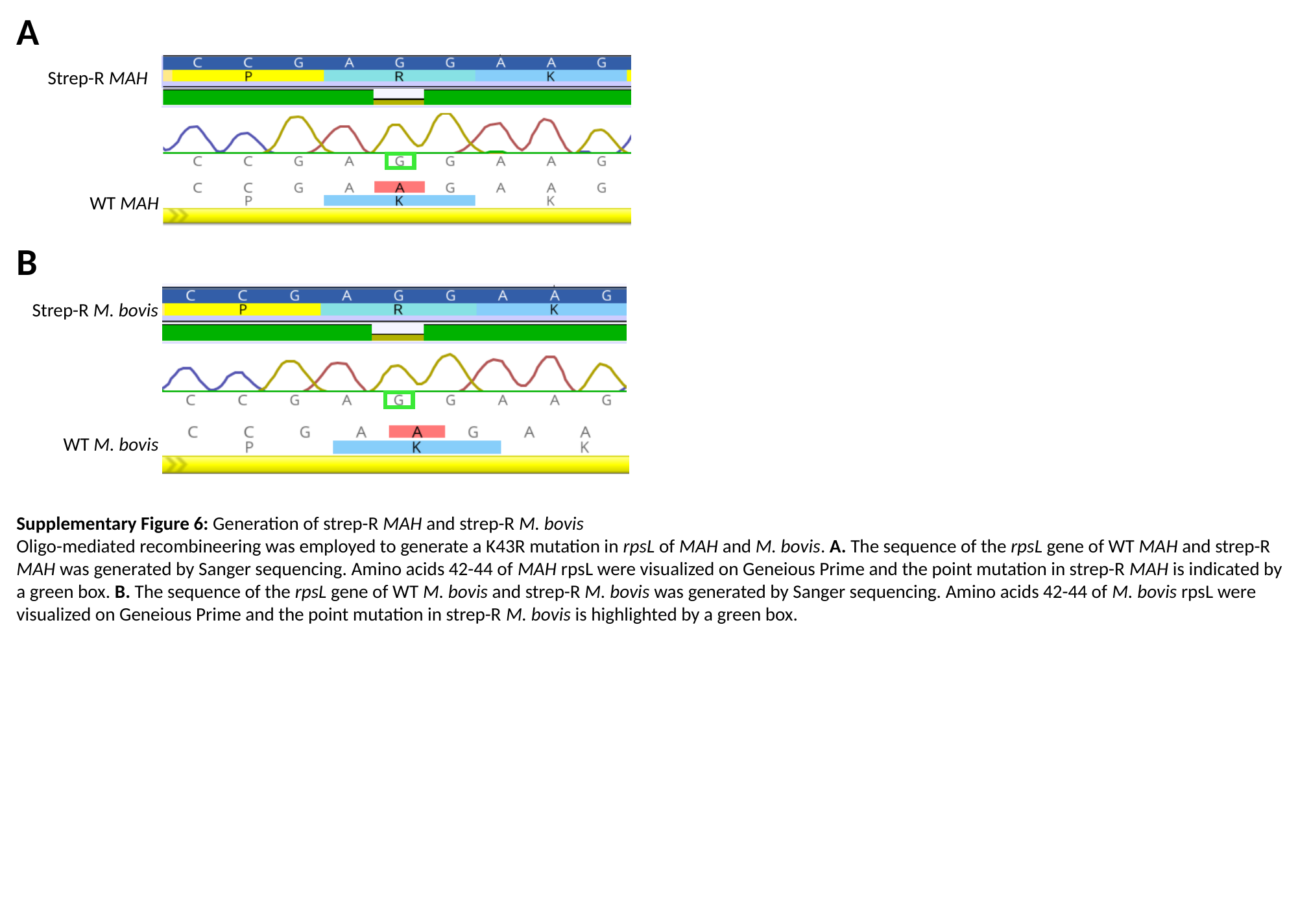

A
Strep-R MAH
WT MAH
B
Strep-R M. bovis
WT M. bovis
Supplementary Figure 6: Generation of strep-R MAH and strep-R M. bovis
Oligo-mediated recombineering was employed to generate a K43R mutation in rpsL of MAH and M. bovis. A. The sequence of the rpsL gene of WT MAH and strep-R MAH was generated by Sanger sequencing. Amino acids 42-44 of MAH rpsL were visualized on Geneious Prime and the point mutation in strep-R MAH is indicated by a green box. B. The sequence of the rpsL gene of WT M. bovis and strep-R M. bovis was generated by Sanger sequencing. Amino acids 42-44 of M. bovis rpsL were visualized on Geneious Prime and the point mutation in strep-R M. bovis is highlighted by a green box.

## Slide 8
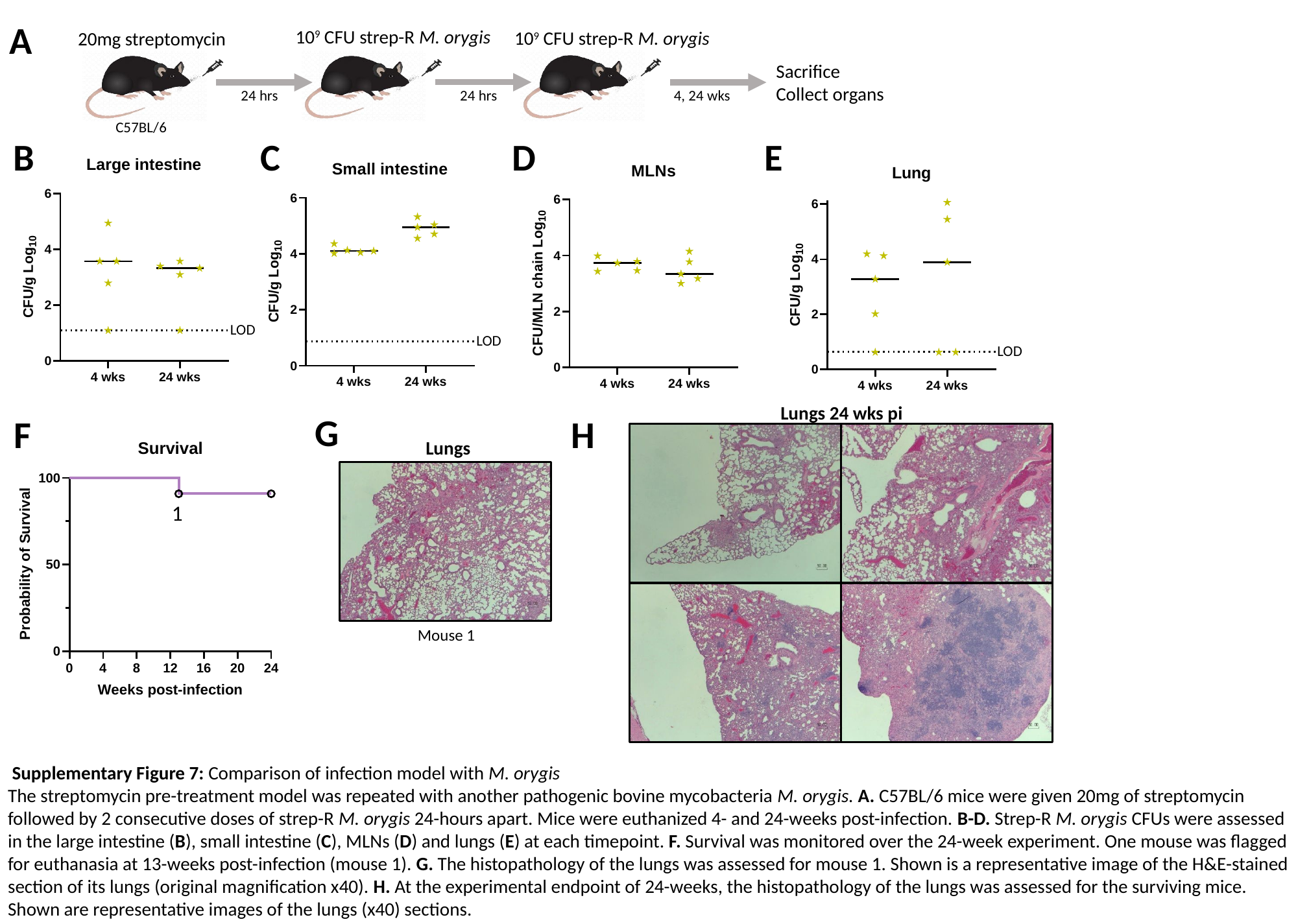

A
109 CFU strep-R M. orygis
109 CFU strep-R M. orygis
20mg streptomycin
Sacrifice
Collect organs
24 hrs
24 hrs
4, 24 wks
C57BL/6
B
C
D
E
LOD
LOD
LOD
Lungs 24 wks pi
G
F
H
Lungs
1
Mouse 1
 Supplementary Figure 7: Comparison of infection model with M. orygis
The streptomycin pre-treatment model was repeated with another pathogenic bovine mycobacteria M. orygis. A. C57BL/6 mice were given 20mg of streptomycin followed by 2 consecutive doses of strep-R M. orygis 24-hours apart. Mice were euthanized 4- and 24-weeks post-infection. B-D. Strep-R M. orygis CFUs were assessed in the large intestine (B), small intestine (C), MLNs (D) and lungs (E) at each timepoint. F. Survival was monitored over the 24-week experiment. One mouse was flagged for euthanasia at 13-weeks post-infection (mouse 1). G. The histopathology of the lungs was assessed for mouse 1. Shown is a representative image of the H&E-stained section of its lungs (original magnification x40). H. At the experimental endpoint of 24-weeks, the histopathology of the lungs was assessed for the surviving mice. Shown are representative images of the lungs (x40) sections.
