# Supplementary figures and images for: Correction: Establishment of persistent enteric mycobacterial infection following streptomycin pre-treatment
Source: Gut Pathog. 2024 Sep 29;16:54. doi: 10.1186/s13099-024-00649-1 (PMC11441166; doi:10.1186/s13099-024-00649-1)

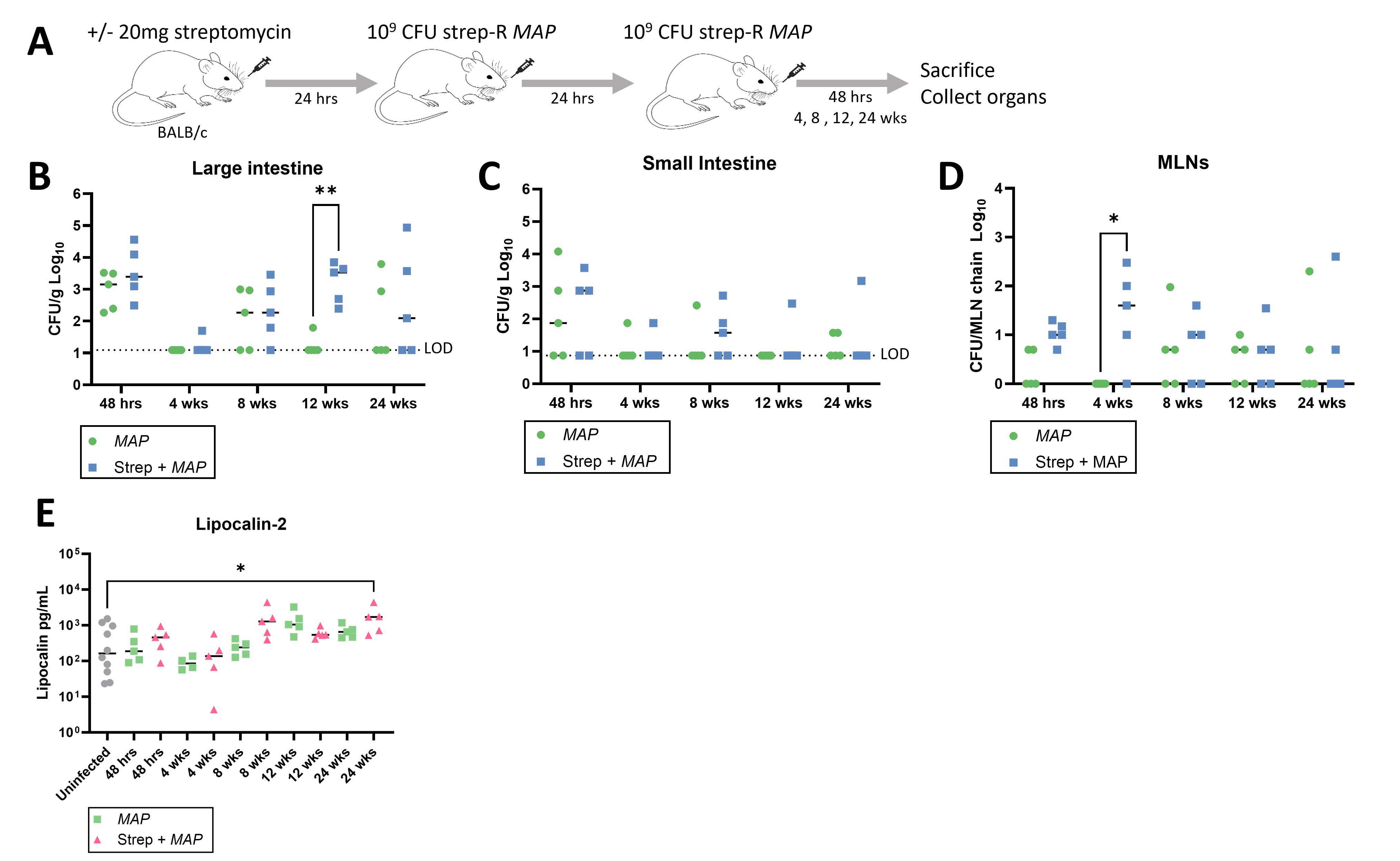

Supplement: Supplementary file 1 — Supplementary Material 1 [file 13099_2024_649_MOESM1_ESM.tif]
